# Exploring antimicrobial resistance to beta-lactams, aminoglycosides and fluoroquinolones in *E. coli* and *K. pneumoniae* using proteogenomics

**DOI:** 10.1038/s41598-021-91905-w

**Published:** 2021-06-14

**Authors:** Dimard E. Foudraine, Nikolaos Strepis, Christoph Stingl, Marian T. ten Kate, Annelies Verbon, Corné H. W. Klaassen, Wil H. F. Goessens, Theo M. Luider, Lennard J. M. Dekker

**Affiliations:** 1 Department of Medical Microbiology and Infectious Diseases, Erasmus University Medical Center Rotterdam, Dr. Molewaterplein 40, 3015 GD Rotterdam, The Netherlands; 2 Department of Neurology, Neuro-Oncology Laboratory/Clinical and Cancer Proteomics, Erasmus University Medical Center Rotterdam, Rotterdam, The Netherlands

**Keywords:** Antimicrobial resistance, Proteomics

## Abstract

Antimicrobial resistance is mostly studied by means of phenotypic growth inhibition determinations, in combination with PCR confirmations or further characterization by means of whole genome sequencing (WGS). However, the actual proteins that cause resistance such as enzymes and a lack of porins cannot be detected by these methods. Improvements in liquid chromatography (LC) and mass spectrometry (MS) enabled easier and more comprehensive proteome analysis. In the current study, susceptibility testing, WGS and MS are combined into a multi-omics approach to analyze resistance against frequently used antibiotics within the beta-lactam, aminoglycoside and fluoroquinolone group in *E. coli* and *K. pneumoniae*. Our aim was to study which currently known mechanisms of resistance can be detected at the protein level using liquid chromatography–mass spectrometry (LC–MS/MS) and to assess whether these could explain beta-lactam, aminoglycoside, and fluoroquinolone resistance in the studied isolates. Furthermore, we aimed to identify significant protein to resistance correlations which have not yet been described before and to correlate the abundance of different porins in relation to resistance to different classes of antibiotics. Whole genome sequencing, high-resolution LC–MS/MS and antimicrobial susceptibility testing by broth microdilution were performed for 187 clinical *E. coli* and *K. pneumoniae* isolates. Resistance genes and proteins were identified using the Comprehensive Antibiotic Resistance Database (CARD). All proteins were annotated using the NCBI RefSeq database and Prokka. Proteins of small spectrum beta-lactamases, extended spectrum beta-lactamases, AmpC beta-lactamases, carbapenemases, and proteins of 16S ribosomal RNA methyltransferases and aminoglycoside acetyltransferases can be detected in *E. coli* and *K. pneumoniae* by LC–MS/MS. The detected mechanisms matched with the phenotype in the majority of isolates. Differences in the abundance and the primary structure of other proteins such as porins also correlated with resistance. LC–MS/MS is a different and complementary method which can be used to characterize antimicrobial resistance in detail as not only the primary resistance causing mechanisms are detected, but also secondary enhancing resistance mechanisms.

## Introduction

Antimicrobial resistance (AMR) is widely recognized as a serious threat to global public health^1^. Among drug resistant pathogens, antibiotic resistant *Escherichia coli* and *Klebsiella pneumoniae* are particularly worrisome. Both species belong to the family of *Enterobacterales* and are clinically relevant as they frequently cause various infections in patients of all ages. Infections caused by these bacteria include urinary tract infection, cholangitis and sepsis^2,3^. Both organisms have become increasingly resistant to several antibiotics. A recent study estimated that third-generation cephalosporin-resistant *E. coli* and *K. pneumoniae* were responsible for approximately 300,000 and 70,000 infections in the European Economic Area in 2015^4^. Furthermore, the WHO considers the development of new antibiotics critical to combat carbapenem resistant and extended-spectrum beta-lactamase (ESBL) producing *Enterobacterales*^5^.

To better diagnose and treat antibiotic resistant micro-organisms, a thorough understanding of AMR-mechanisms is paramount. In the last three decades, our understanding of AMR-mechanisms has increased significantly by unravelling their genome using DNA sequencing. Currently, whole genome sequencing (WGS) has advanced to a point where complete bacterial genomes can be sequenced within hours. The genetic bases of many AMR-mechanisms are known, and both resistance and susceptibility to some antibiotics can be predicted for important pathogens using WGS^6–8^.

However, the detection of genes encoding resistance mechanisms does not provide information on the subsequent transcription and translation processes resulting in different protein quantities and enzymatic activity, and it does also not provide information on the interaction between different resistance mechanisms, e.g., a decrease in porins combined with increased beta-lactamase production^9–11^. One of the ways to detect and assess the abundance of these mechanisms is by analysis of the bacterial proteome. Similar to the achievements made for WGS, liquid chromatography combined with mass spectrometry (LC–MS) has advanced to a level in which proteomes can be comprehensively characterized within a few hours using bottom-up shotgun proteomics^12^. Both Chang et al. and Trip et al. applied this discovery-based approach to detect beta-lactamases in several *Acinetobacter baumannii* isolates^13,14^. In addition to discovery-based approaches which are mainly used in a research setting, targeted protein detection methods are being developed that show potential for diagnostic testing^9,15,16^. Besides being highly accurate, these methods offer a shorter turnaround time than the currently applied phenotypical susceptibility testing techniques that require overnight incubation which results in a delay in reporting time^17^.

In the current study, we analyzed which antimicrobial resistance genes were detected at the protein level using LC–MS/MS without applying antibiotic pressure. The detected genes and their respective proteins were subsequently matched with the susceptibility results obtained for beta-lactam, aminoglycoside, and fluoroquinolone antibiotics. Furthermore, we studied the relationship between the abundance of different porins and whether this correlated to resistance against different classes of antibiotics. Finally, we identified protein to resistance correlations that have not yet been described before. For these objectives, data-dependent acquisition (DDA) LC–MS/MS, WGS and antimicrobial susceptibility testing (AST) were performed for a selected set of 187 *E. coli* and *K. pneumoniae* isolates containing various antibiotic resistance mechanisms.

## Results

### Sample characteristics

**Figure 1:**
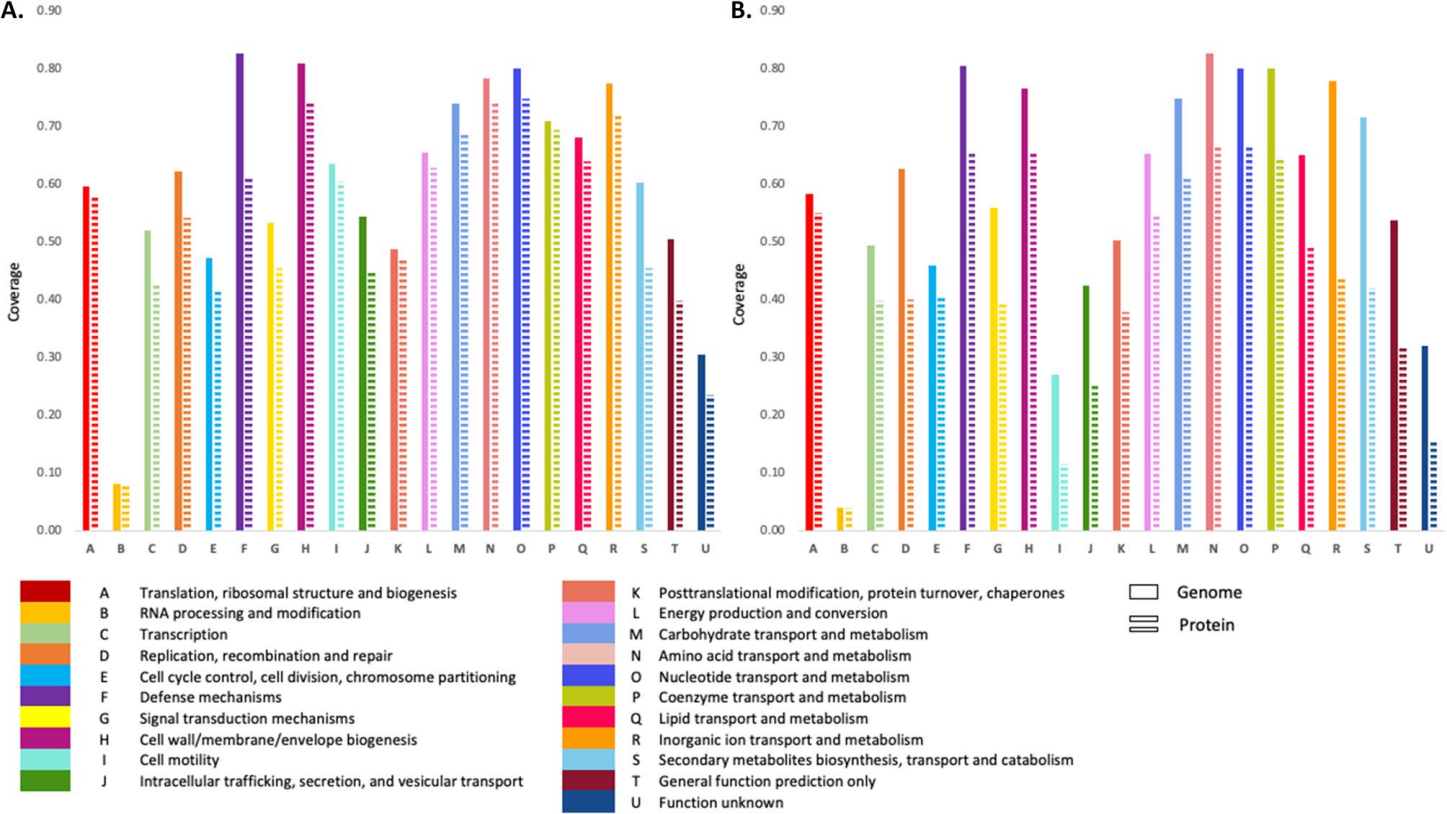
Clusters of orthologous groups (COG) analysis of all genes and all proteins which were detected at least once in the *E. coli* isolates (**A**) and in the *K. pneumoniae* isolates (**B**). X-axis: The major functional COG families. Y-axis: The proportion of genes and proteins that were detected in both species compared to the COG database. Genes are represented by solid bars and proteins are represented by interrupted bars.

In the current study, 187 *E. coli* and *K pneumoniae* isolates were included displaying a wide variety of beta-lactam, aminoglycoside and quinolone resistance mechanisms. We included isolates containing small spectrum and extended spectrum beta-lactamases, carbapenemases and isolates containing different aminoglycoside modifying enzymes as well as 16S rRNA methyltransferases. Before analyzing resistance mechanisms, we first determined to which extent high-resolution mass spectrometry was capable of detecting and characterizing the proteome of the *E. coli* and *K. pneumoniae* isolates in comparison to the information obtained by WGS. For this purpose, clusters of orthologous groups of proteins (COG) analysis was performed^18^. In Fig. 1, the correlation of all proteins and genes that were detected at least once are shown for the major functional families. Proteins were detected in each functional family of which genes were detected with none of the families showing a particular low protein to gene ratio. On average 2174 (± 56) proteins were detected per *E. coli* isolate and 2302 (± 69) proteins per *K. pneumonia* isolate. This corresponded with an average predicted protein coverage by LC–MS/MS of 0.46 in *E. coli* (4747 ± 230 predicted proteins) and 0.44 in *K. pneumonia* (5199 ± 188 predicted proteins).

**Figure 2:**
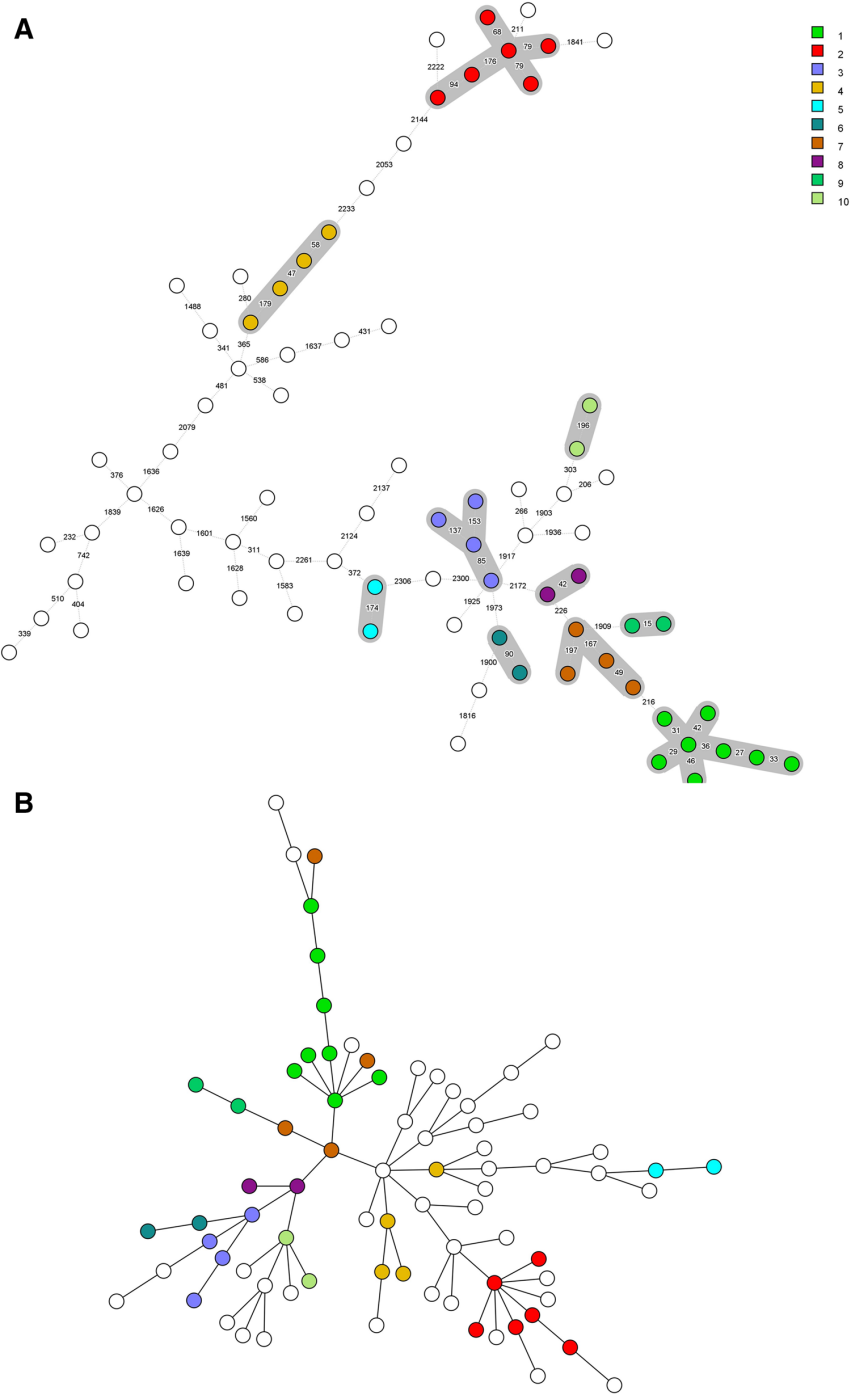

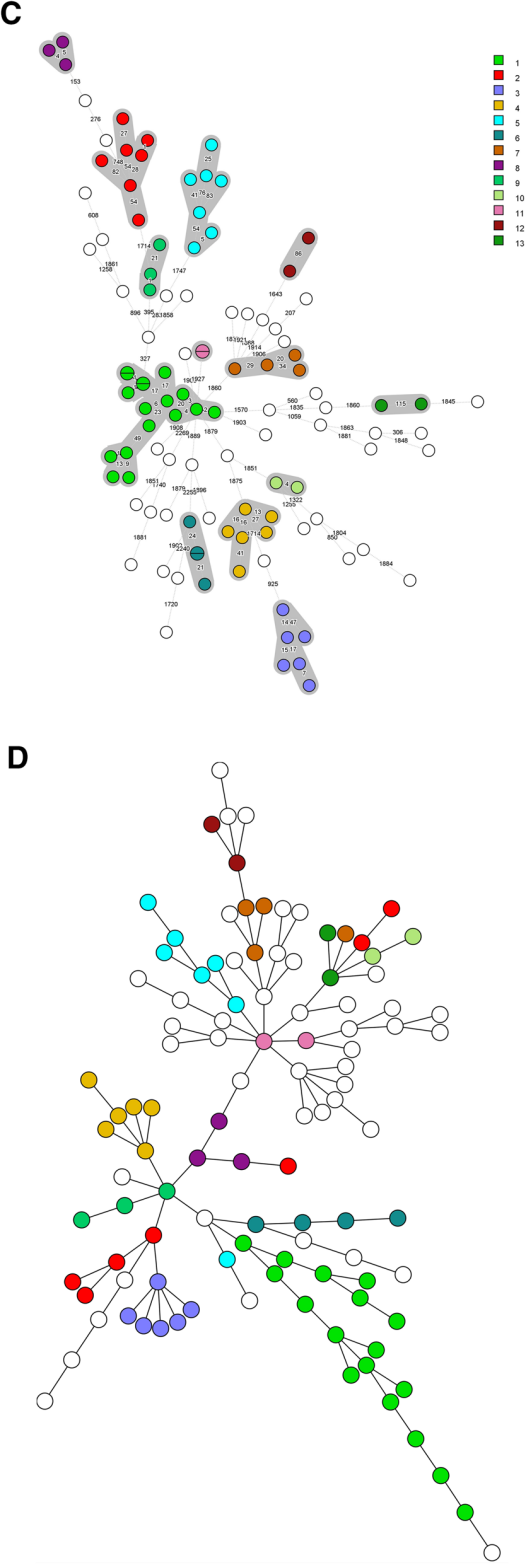
A comparison of sample clustering based on core genome multilocus sequence typing (cgMLST) and clustering based on protein intensity. Each circle represents one isolate. (**A**, **B**) Display the clustering obtained by cgMLST and protein intensity analysis for the 78 *E. coli* isolates. (**C**, **D**) Display the clustering obtained by cgMLST and protein intensity analysis for the 109 K*. pneumoniae* isolates. Each cluster was indicated with a color and numbered. The same isolates were colored in both the cgMLST and protein intensity analysis.

To demonstrate the heterogeneity of the selected isolates harboring different resistance mechanisms, clustering based on core genome multilocus sequence typing (cgMLST) and clustering based on protein intensity were compared. For *E. coli*, both the cgMLST as well as clustering based on protein intensity showed a diverse set of isolates with some clustering but all isolates differed by at least 15 alleles. The *K. pneumoniae* isolates were also diverse but several clusters of isolates were present. In general, both cgMLST and protein intensity analysis showed similar clustering of isolates (Fig. 2).

### Beta-lactam resistance

**Figure 3:**
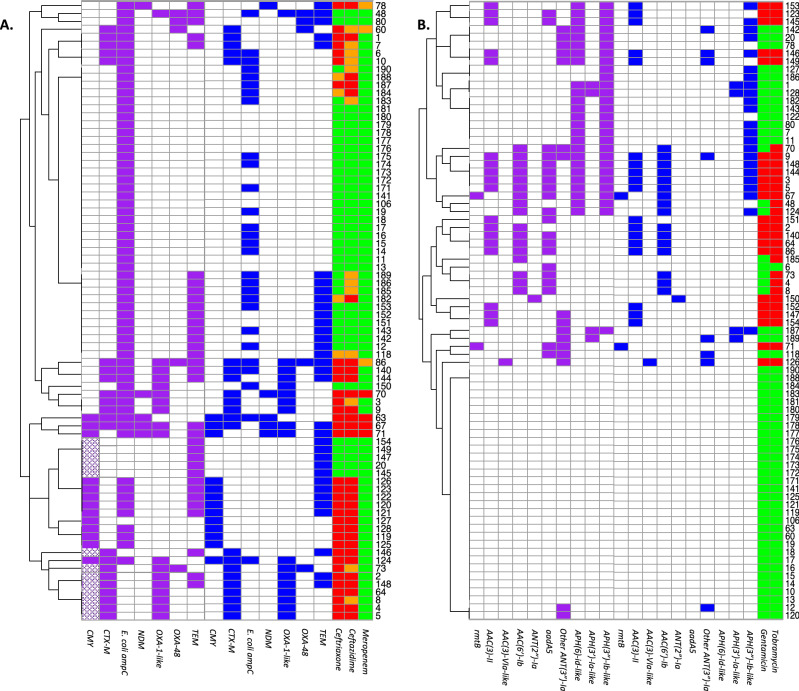

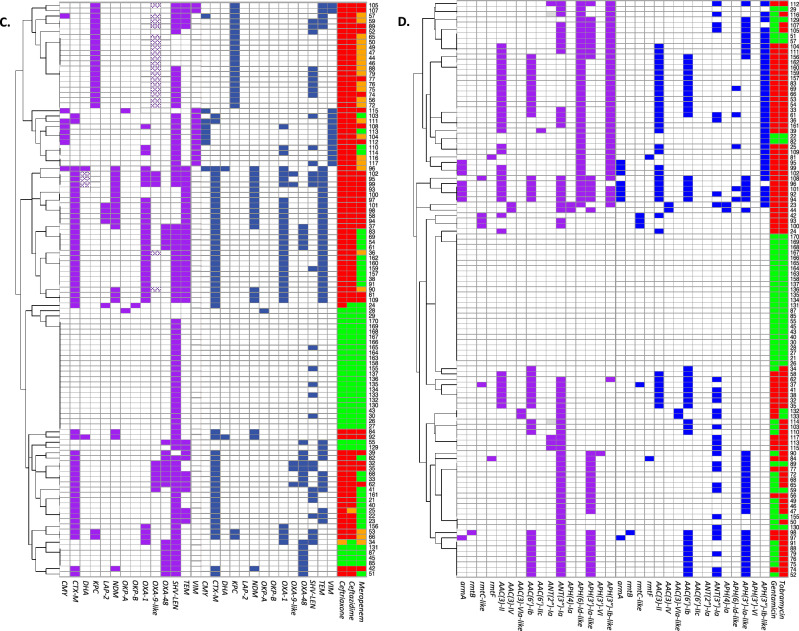
An overview of the different resistance mechanisms at the gene and protein level in the studied *E. coli* and *K. pneumoniae* isolates. The presence of beta-lactamase genes and proteins in the 78 *E. coli* and 109 K*. pneumoniae* isolates are shown in (**A**, **C**). The presence of 16S ribosomal RNA methyltransferase (16S-RMTase) and aminoglycoside modifying enzyme (AME) genes and proteins are shown in (**B**, **D**) for *E. coli* and *K. pneumoniae*, respectively. For each isolate, present genes were depicted in purple, proteins in blue and susceptible, intermediate and resistant phenotypes in green, orange, and red, respectively. Genes that were partly present and genes that were less than 80% similar to their reference genes were depicted with a dotted pattern. Isolates were clustered based on presence of the depicted AMR genes.

The carbapenemases NDM, OXA-48, KPC and VIM were detected at the protein level in all isolates carrying the corresponding genes. Similar to these carbapenemases, the extended spectrum beta-lactamase (ESBL) CTX-M and the beta-lactamases TEM and OXA-1 (Tables 1, 2 and Fig. 3) were also detected in all isolates carrying corresponding genes. Some of the other beta-lactamases were not always detected with LC–MS/MS. This was the case for SHV/LEN which was only detected in 30 of the 91 K*. pneumoniae* isolates with an encoding gene. Furthermore, presence of *bla*_CMY-132_-like genes in 13 *E. coli* isolates, and presence of *bla*_DHA_ and *bla*_OXA-9_-like genes in 3 and 20 K*. pneumoniae* isolates was predicted by the resistance gene identifier, however, the corresponding proteins were not detected with MS. Additional analysis of WGS data showed that these genes were either partially present or were less than 80% similar to their reference genes. The proteins of the beta-lactamases OKP-B and LAP-2 were not detected in any of the isolates containing these genes. A peculiar result was obtained for the chromosomally encoded *bla*_AmpC_ in *E. coli*. It is generally accepted that all *E. coli* isolates carry a chromosomally encoded *bla*_AmpC_ gene which is in most cases not expressed or minimally expressed^19,20^. However, in our selected isolates, *bla*_AmpC_ genes were only detected in 64 of the 78 *E. coli* isolates. In 28 of these 64 isolates, AmpC proteins were detected as well.

**Table 1:** Presence of genes and proteins of beta-lactamases, 16S ribosomal RNA methyltransferases, aminoglycoside modifying enzymes and quinolone resistance proteins in the 78 analysed *E. coli*.

	Number of isolates with AMR gene	Number of isolates with AMR gene and protein	Proportion of isolates in which AMR gene was detected at the protein level (%)
CMY^a^	26	13	50
CTX-M	22	22	100
*Escherichia coli* ampC	64	28	44
NDM	5	5	100
OXA-1-like	18	18	100
OXA-48	5	5	100
TEM	34	34	100
RmtB	2	2	100
AAC(3)-II	18	18	100
AAC(3)-VIa-like	1	1	100
AAC(6′)-Ib	17	16	94
ANT(2″)-Ia	1	1	100
aadA5^b^	20	0	0
Other ANT(3″)-Ia	16	8	50
APH(6)-Id-like	25	0	0
APH(3′)-Ia-like	4	4	100
APH(3″)-Ib-like	28	23	82
QnrS1	2	0	0

Origin and nomenclature of beta-lactamase resistance genes and aminoglycoside modifying enzymes are described in the publication of Jacoby, and the publication of Ramirez and Tolmasky, respectively^21,22^.

^a^Presence of a CMY-132-like gene was predicted in 13 isolates using the Resistance Gene Identifier. However, these genes had less than 80% similarity to CMY-132. Furthermore, a CMY protein was not detected by MS in any of these 13 isolates.

^b^Although aadA5 also belongs to the ANT(3″)-Ia group, the protein sequence is quite distinct from the other ANT(3″)-Ia enzymes detected in this study. Therefore, the distinction between aadA5 and other ANT(3″)-Ia enzymes was made.

**Table 2:** Presence of genes and proteins of beta-lactamases, 16S ribosomal RNA methyltransferases, aminoglycoside modifying enzymes, quinolone resistance proteins and OqxAB efflux pumps in the 109 analysed *K. pneumoniae.*

	Number of isolates with AMR mechanism detected in genome	Number of isolates with AMR mechanism in both genome and proteome	Proportion of isolates in which AMR gene was detected at the protein level (%)
CMY	8	8	100
CTX-M	50	50	100
DHA^a^	5	2	40
KPC	22	22	100
LAP-2	1	0	0
NDM	18	18	100
OKP-A	2	1	50
OKP-B	5	0	0
OXA-1	31	31	100
OXA-9-like^b^	29	5	17
OXA-48	20	20	100
SHV/LEN	91	30	33
TEM	42	42	100
VIM	13	13	100
ArmA	8	8	100
RmtB	1	1	100
RmtC-like	5	4	80
RmtF	2	2	100
AAC(3)-II	35	35	100
AAC(3)-IV	2	2	100
AAC(3)-VIa-like	2	2	100
AAC(6′)-Ib	37	37	100
AAC(6′)-IIc	1	0	0
ANT(2″)-Ia	4	0	0
ANT(3″)-Ia	60	30	50
APH(4)-Ia	2	2	100
APH(6)-Id-like	39	5	13
APH(3′)-Ia-like	36	36	100
APH(3′)-VI	1	0	0
APH(3″)-Ib-like	39	36	92
QnrA1	5	5	100
QnrB	16	11	69
QnrS	8	0	0
OqxA	108	32	30
OqxB	107	32	30

Origin and nomenclature of beta-lactamase resistance genes and aminoglycoside modifying enzymes are described in the publication of Jacoby, and the publication of Ramirez and Tolmasky, respectively^21,22^.

^a^DHA genes were detected in five isolates. However, in three of these isolates only 70% of the sequence was covered. The corresponding proteins were not detected by MS.

^b^OXA-9-like genes were detected in 29 isolates. However in 20 of these isolates only 40% of the sequence was covered. The corresponding protein were not detected by MS in any of these 20 isolates but also not in four of the nine isolates with a completely covered gene.

To correlate the presence of the various beta-lactamases with their phenotypes, we classified isolates arbitrarily based on their MICs for 3rd generation cephalosporins and meropenem into “wild type” (WT) combined with small-spectrum beta-lactamase and/or oxacillinase-producing isolates (PEN/OXA), ESBL or AmpC-producing isolates, chromosomal *E. coli* AmpC-producing isolates (for *E. coli* only), or carbapenemase producing isolates (CPE). For *E. coli*, the isolates susceptible to 3rd generation cephalosporins and with meropenem MICs below the epidemiological cut-off value (ECOFF; www.eucast.org) of 0.125 mg/L did either not have any acquired beta-lactamases (n = 21) or had a small spectrum beta-lactamase such as TEM (n = 11) or OXA-1 (n = 1). Remarkably, in 12 of these 33 3rd generation susceptible isolates, the chromosomally encoded AmpC enzyme was still detected at the protein level without compromising the susceptibility to ceftazidime or ceftriaxone. In the isolates that were not susceptible to ceftazidime or ceftriaxone but with meropenem MICs ≤ 0.125 mg/L (n = 35), the ESBL CTX-M (n = 16), a CMY enzyme (n = 10) or only a chromosomal AmpC (n = 9) were detected. In the nine *E. coli* isolates in which only chromosomal AmpC proteins were detected, this resulted in ceftazidime MICs of 4–8 mg/L and ceftriaxone MICs < 2 mg/L except for one isolate with a ceftriaxone MIC of 4 mg/L. In the ten meropenem-resistant *E. coli* isolates (CPE) either OXA-48 or NDM proteins were detected, sometimes in combination with CMY and/or CTX-M (Table 3). Remarkably, in two OXA-48-positive isolates with an MIC of 8 mg/L for meropenem, the porin OmpC could not be demonstrated while in the remaining three isolates with MICs of 0.5–2 mg/L, OmpC was detected. This suggests a difference in OmpC abundance which may explain this difference in meropenem MICs.

**Table 3:** Seventy-eight *E. coli* isolates classified according to their beta-lactamase phenotype including the detected resistance mechanisms against beta-lactams and aminoglycosides by LC–MS/MS.

Phenotype	n	Antibiotics						PEN/OXA		ESBL	AmpC		CPE		16S-RMTase	AME			
		CRX	CAZ	MEM	GEN	TOB	CIP	TEM	OXA-1	CTX-M	CMY	cAmpC	OXA-48	NDM	RmtB	AAC(3)-II	AAC(3)-VI	AAC(6′)-Ib	ANT(2″)-Ia
WT/PEN	25	S	S	S	S	S	S/R	4	–	–	–	10	–	–	–	–	–	–	–
WT/PEN/AME	8	S	S	S	R	R	S/R	7	1	–	–	2	–	–	–	7	–	–	1
ESBL/AmpC	12	S/R	S/R	S	S	S	S/R	6	–	4	7	2	–	–	–	–	–	–	–
ESBL/AmpC/AME	14	R	R	S	S/R	R	S/R	7	11	12	3	2	–	–	–	10	1	11	–
AmpC	6	S/R	R	S	S	S	S	1	–	–	–	6	–	–	–	–	–	–	–
AmpC/Quin	3	S	R	S	S	S/R	R	3	–	–	–	3	–	–	–	–	–	–	–
CPE	3	S/R	S/R	S/R	S	S	S	2	–	1	–	–	2	1	–	–	–	–	–
CPE/Quin	7	S/R	S/R	S/R	S/R	S/R	R	4	6	5	3	3	3	4	2	1	–	5	–

Isolates were classified based on their susceptibility (S) or resistance (R) to ceftriaxone (CRX), ceftazidime (CAZ), meropenem (MEM), gentamicin (GEN), tobramycin (TOB) and ciprofloxacin (CIP) into wild type (WT) isolates and small spectrum penicillinase (PEN) and/or oxacillinase-producing (OXA) isolates, extended-spectrum beta-lactamase producers (ESBL) in combination with isolates producing AmpC beta-lactamases (AmpC), isolates that only produced *E. coli* chromosomal AmpC, or carbapenemase producing *Enterobacterales* (CPE). Isolates were further divided based on aminoglycoside resistance, mostly caused by aminoglycoside modifying enzymes (AME), and/or quinolone resistance (Quin). Small spectrum beta-lactamases/oxacillinases included TEM and OXA-1. ESBL and AmpC were represented by CTX-M, CMY and *E. coli* chromosomal AmpC. Carbapenemase producing *Enterobacterales* (CPE) included OXA-48 and New Delhi Metallo beta-lactamase (NDM) producing isolates. Mechanisms of aminoglycoside resistance included the 16S rRNA methyltransferase (rmtB), the acetyltransferases AAC(3)-II, AAC(3)-VI and AAC(6′)-Ib, and the nucleotidyltransferase ANT(2″)-I.

For the *K. pneumoniae* isolates, a similar analysis was performed. We tried to explain the different phenotypes by analyzing the beta-lactamases detected at the protein level (Table 4). In all 29 isolates susceptible to 3rd generation cephalosporins, no enzymes were detected with substrate specificity to 3rd generation cephalosporins except for one isolate in which the ESBL SHV-27 was detected. This isolate still had MICs of 0.25 mg/L or lower for ceftazidime and ceftriaxone. Of the 3rd generation cephalosporin susceptible isolates, five isolates had meropenem MICs of 0.25–1 mg/L and were positive for OXA-48 at both the genome and the proteome level. In the group of 3rd generation cephalosporin resistant isolates with meropenem MIC’s ≤ 0.25 mg/L, CTX-M was detected (n = 12). In addition, in some of these isolates TEM-1 and OXA-1 were also present. The remaining group consisting of 68 isolates all displayed MICs above the screening breakpoint for meropenem and in the majority of these isolates, one carbapenemase was demonstrated per isolate. The isolates in which either KPC or VIM was detected showed a wide range of MICs suggesting that additional resistance mechanisms are acting in concert to affect the meropenem MICs. The isolates in which OXA-48 enzymes were detected also showed a wide range in meropenem MICs. Nevertheless, all OXA-48 enzymes were detected by LC–MS/MS. In one isolate with an MIC of 4 mg/L for meropenem, no carbapenemases were detected but instead CTX-M, TEM and OXA-1 were demonstrated while the two major porins OmpK35 and OmpK36 were not detected at the protein level. The combination of these mechanisms may explain the increased MIC to meropenem. In general, LC–MS/MS was able to demonstrate the presence of the proteins conferring resistance to meropenem and 3rd generation cephalosporins, even in organisms harboring mechanisms that were hard to detect by phenotypic assays, i.e. in isolates displaying low MICs for their indicator antibiotics.

**Table 4:** One hundred and nine *K. pneumoniae* isolates classified according to their beta-lactamase phenotype including the detected resistance mechanisms against beta-lactams and aminoglycosides by LC–MS/MS.

Phenotype	n	Antibiotics						PEN/OXA				ESBL	AmpC		CPE				16S-RMTase	AAC	
		CRX	CAZ	MEM	GEN	TOB	CIP	TEM	SHV	OXA-1	OXA-9	CTX-M	CMY	DHA	OXA-48	NDM	KPC	VIM		AAC(3)	AAC(6′)
WT/PEN/OXA	27	S	S	S	S	S	S/R	2	4	–	–	–	–	–	5	–	–	–	–	–	–
WT/PEN/OXA/AME	2	S	S	S	R	S	S	–	1	–	–	–	–	–	–	–	–	–	–	2	–
ESBL/AmpC	2	R	R	S	S	S	S/R	1	2	–	–	2	–	–	–	–	–	–	–	–	–
ESBL/AmpC/AME	10	R	S/R	S	R	S/R	S/R	7	2	6	–	10	–	–	–	–	–	–	–	9	6
CRE/Quin	4	R	R	S/R	S	S	R	3	2	–	–	1	1	–	–	–	3	–	–	–	–
CRE/AME	6	R	R	S/R	S/R	S/R	S/R	1	1	2	–	2	1	–	2	–	–	4	–	–	2
CRE/AME/Quin	58	R	R	S/R	S/R	R	R	28	18	23	5	35	6	2	13	18	19	9	15	28	29

Isolates were classified based on their susceptibility (S) or resistance (R) to ceftriaxone (CRX), ceftazidime (CAZ), meropenem (MEM), gentamicin (GEN), tobramycin (TOB) and ciprofloxacin (CIP) into wild type (WT) isolates and small spectrum penicillinase (PEN) and/or oxacillinase-producing (OXA) isolates, extended-spectrum beta-lactamase producers (ESBL) in combination with isolates producing AmpC beta-lactamases (AmpC), or carbapenem resistant *Enterobacterales* (CRE). All CRE isolates had MEM MIC’s ≥ 0.5 mg/L. Isolates were further divided based on aminoglycoside resistance, mostly caused by aminoglycoside modifying enzymes (AME) and/or quinolone resistance (Quin). Small spectrum beta-lactamases/oxacillinases included TEM, SHV, OXA-1 and OXA-9. ESBL and AmpC were represented by CTX-M, CMY and DHA. Carbapenemase producing *Enterobacterales* (CPE) included OXA-48, NDM, KPC and VIM positive isolates. Mechanisms of aminoglycoside resistance included the 16S rRNA methyltransferases ArmA, RmtB, RmtC and RmtF and the acetyltransferase group AAC(3) which included AAC(3)-II, AAC(3)-IV and AAC(3)-VI and the acetyltransferase AAC(6′)-Ib.

### Aminoglycoside resistance

Aminoglycoside resistance in *Enterobacterales* is mainly caused by aminoglycoside modifying enzymes (AMEs) and/or the presence of 16S ribosomal RNA methyltransferases (16S-RMTases)^21,23^. In the examined isolates, genes encoding four different 16S-RMTases and 12 different AME groups were detected. All 16S-RMTase genes, i.e. *armA*, *rmtB*, *rmtC* and *rmtF* were detected at the protein level by MS except for *rmtC* in one isolate. Of the AMEs, most acetyltransferases (AAC) were detected by MS while this varied for adenyltransferases (ANT) and phosphotransferases (APH). Results for all 16S-RMTases and AMEs are shown in Tables 1, 2 and Fig. 3.

Previous studies have shown major differences in substrate specificity between the different aminoglycoside modifying enzymes^21^. This was also confirmed in the present study as for instance the presence of only ANT(3″)-Ia, APH(6)-Id-like, or APH (3″)-Ib-like enzymes in *E. coli* did not result in resistance to gentamicin or tobramycin. In contrast, the 18 isolates with AAC(3)-II, the isolate with AAC(3)-VIa-like and the isolate with ANT(2″)-Ia, all showed MICs > 8 mg/L for gentamicin and MICs ≥ 4 mg/L for tobramycin, indicating resistance to both antibiotics. Furthermore, the 16 isolates with only AAC(6′)-Ib all had MICs ≥ 8 mg/L for tobramycin and MICs of 1 or 2 mg/L for gentamicin. In one isolate an *AAC(6*′*)-Ib9-like* gene resulted in an MIC of 8 mg/L for tobramycin but the corresponding protein was not detected. The two isolates in which the 16S-RMTase RmtB was detected had MICs > 8 mg/L for both gentamicin and tobramycin. All 49 remaining isolates with none of these resistance mechanisms were susceptible to gentamicin and tobramycin.

For *K. pneumoniae*, generally similar observations were made as for *E. coli* (Table 4). All *K. pneumoniae* isolates in which a 16S-RMTase (n = 16) was detected had MICs > 8 mg/L for both gentamicin and tobramycin. All isolates in which an AAC(3) enzyme (n = 39) was detected had MICs > 8 mg/L for gentamicin and usually also MICs > 8 mg/L for tobramycin (n = 34). However, in many of these isolates AAC(6′)-Ib was detected as well. In absence of other AACs or 16S-RMTases, the presence of only AAC(6′)-Ib (n = 11) resulted in MICs > 8 mg/L for tobramycin and MICs ≤ 2 mg/L for gentamicin, except for one isolate with a gentamicin MIC of 4 mg/L. In 4 isolates an *ant(2″)-Ia* gene was identified which conferred resistance to both gentamicin and tobramycin. However, the corresponding protein was not detected. In 12 of the 72 K*. pneumoniae* isolates resistant to tobramycin, no AME or 16S-RMTase genes were identified that could explain the corresponding phenotype. However in 9 of these 12 isolates, AAC(6′)-Ib(-like) proteins were detected. This indicated the presence of this enzyme or a similar protein which most likely explains the tobramycin resistance.

### Fluoroquinolone resistance

Fluoroquinolone resistance in *E. coli* and *K. pneumoniae* can be caused by (a combination of) target site mutations, enzymes modifying fluoroquinolones, physical blocking of the target site by Qnr proteins, and presence or increased expression of specific efflux pumps^10,24^.

In the analyzed isolates, QnrA was detected by MS in five out of five isolates with encoding genes and QnrB in 11 out of 16 isolates. Remarkably, QnrS was not detected in any of the ten isolates with encoding genes (Tables 1 and 2). The AME AAC(6′)-Ib-cr which also acetylates ciprofloxacin in addition to aminoglycosides was detected in all isolates (41/41). The genes *oqxA* and *oqxB* were detected in 108 and 107 K*. pneumoniae* isolates, respectively, but in none of the *E. coli* isolates. The OqxAB protein complex was detected in 29 K*. pneumoniae* isolates while only OqxA was detected in three isolates and only OqxB in three other isolates.

We assessed whether the detected fluoroquinolone resistance mechanisms could explain increased MICs to ciprofloxacin. In the 36 *E. coli* isolates with an MIC ≤ the ECOFF of 0.064 mg/L no resistance mechanisms were detected. In contrast, all 33 resistant *E. coli* isolates with an MIC ≥ 1 mg/L had at least one mutation linked to fluoroquinolone resistance in *gyrA*. Furthermore, 29 of these isolates had at least one mutation in *parC*. In addition, in 16 isolates AAC(6′)-Ib-cr was detected, and in 2 isolates a *qnrS* gene was identified. Finally, 9 isolates had an MIC of 0.125 or 0.25 mg/L which was above the ECOFF of 0.064 mg/L but still within the susceptible range. In eight of these isolates at least one mutation in *gyrA* was identified, corresponding with the moderately increased MICs.

Of the 109 K*. pneumoniae* isolates*,* 73 isolates were resistant with an MIC ≥ 1 mg/L. In 62 of these isolates, one or more mutations were identified in *parC* and in 37 isolates one or more mutations in *gyrA*. Furthermore, in 28 isolates the OqxAB complex was detected and in 23 isolates AAC(6′)-Ib-cr. In 15 isolates, a *qnrB* gene was identified, in 7 a *qnrS* gene, and in 5 a *qnrA* gene. Thirty isolates had MICs ≤ ECOFF of 0.125 mg/L and in two of these isolates AAC(6′)-Ib-cr was detected. In the 28 remaining isolates, none of these resistance mechanisms were identified, as was also the case for two isolates with an MIC of 0.25 mg/L. Finally, a *qnrB* gene, a *qnrS* gene, a mutation in *gyrA* and the OqxAB protein were each identified once in the four intermediate (MIC of 0.5 mg/L) isolates, thereby explaining these moderately increased MICs.

### Porin analysis

**Figure 4:**
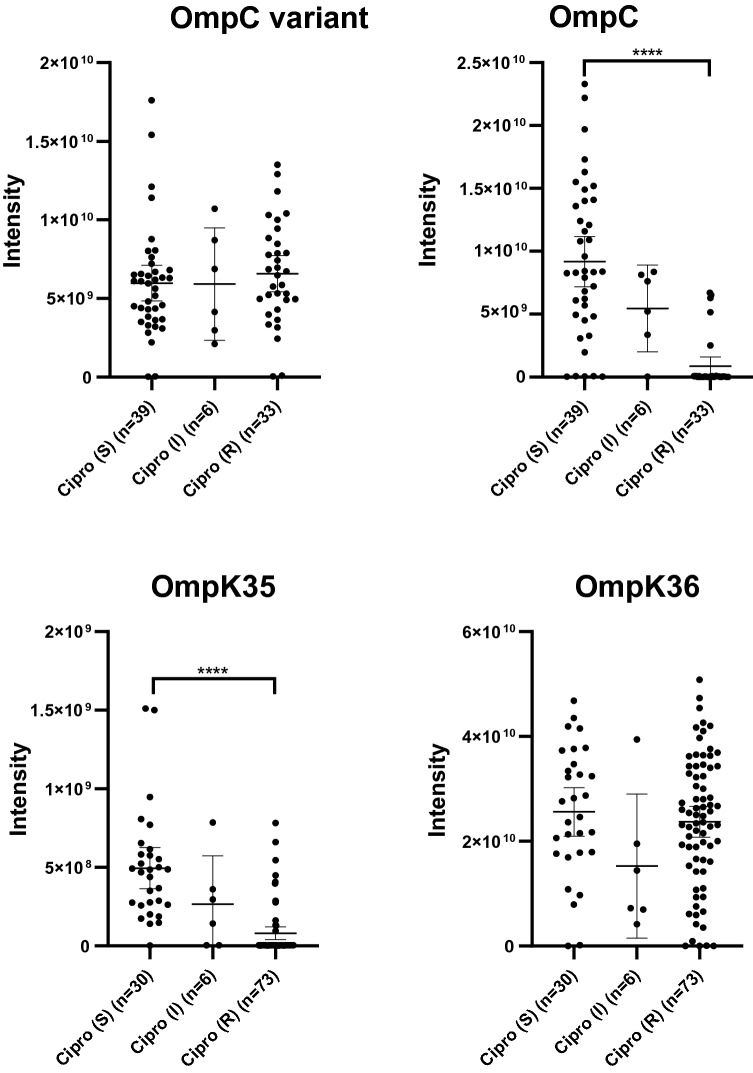
Porin abundance was compared between ciprofloxacin susceptible, intermediate and resistant *E. coli* and *K. pneumoniae* isolates. Signal intensities for the different porins are shown in arbitrary units for the “regular” OmpC and the OmpC variant in *E. coli*, and for OmpK35 and OmpK36 in *K. pneumoniae*. ****p-value < 0.0001 (Ordinary one-way Anova, Turkey’s multiple comparisons test).

The presence of single resistance mechanisms can already cause resistance to certain antibiotic classes. However, secondary mechanisms often contribute to increase MICs past the breakpoint used to determine (non-)susceptibility. For instance, a decrease in porin expression acts in concert with enzymes such as beta-lactamases. In the present study, OmpF abundance in *E. coli* was negatively correlated with increasing MICs to each of the four antibiotic classes studied, but was only found to have a significantly lower abundance in aminoglycoside resistant isolates than in aminoglycoside susceptible isolates. Of the other major porin OmpC, a variant was identified that was significantly less abundant in cephalosporin, aminoglycoside and ciprofloxacin resistant isolates. In contrast, the “regular” OmpC porin was not correlated to resistance (Fig. 4 and Supplementary Fig. 1). Interestingly, the maltoporin LamB was significantly less abundant in meropenem resistant isolates. In *K. pneumoniae,* OmpK35 (orthologue of OmpF) was significantly less abundant in isolates resistant to meropenem, 3rd generation cephalosporins, aminoglycosides or ciprofloxacin. As the majority of these isolates were resistant to more than one class of antibiotics, OmpK35 abundance could not be correlated to resistance against one specific antibiotic class. OmpK36 (orthologue of OmpC) abundance was not significantly correlated to resistance to any of the antibiotics (Fig. 4). The third major porin, i.e. PhoE was not detected in the isolates of either *E. coli* or *K. pneumoniae* which is to be expected as isolates were cultured under general culture conditions without limiting phosphate concentrations.

### Discovery-based analysis

In addition to analysis of specific AMR mechanisms, a discovery-based analysis was performed to identify protein groups which were significantly correlated with resistance to meropenem, third generation cephalosporins, aminoglycosides, ciprofloxacin or a combination of these antibiotics/classes. To compare protein abundance between resistant and susceptible isolates, MS data was log2 transformed and imputation was applied for missing values. As most isolates that were resistant to one antibiotic class were also resistant to other antibiotic classes, most proteins could not be correlated to resistance against one specific class. In the 78 *E. coli* isolates, a total of 7951 protein groups were detected of which 80 groups were significantly more abundant in the resistant isolates. In addition, 46 protein groups were significantly less abundant in the resistant isolates. In the 109 K*. pneumoniae* isolates, 8648 protein groups were detected of which 208 groups were significantly more abundant in the resistant isolates. Furthermore, 82 protein groups were significantly less abundant in the resistant isolates. Data on all detected protein groups including their measured intensities and their correlation to resistance to each of the antibiotic classes is available in the “Supplementary datasets”.

The 46 protein groups which were more than an arbitrary four times as abundant in isolates resistant to any of the antibiotic classes are shown in Tables 5 and 6. Of these, 23 protein groups were already curated as AMR mechanisms and consisted mostly of beta-lactamases or other antibiotic altering or degrading enzymes. The protein groups NDM and CTX-M in *E. coli*, and KPC, CTX-M and AAC(6′)-Ib in *K. pneumoniae* were more than 20 times as abundant in resistant isolates. Some resistance mechanisms correlated more strongly to another antibiotic class than to the class that they primarily affect due to co-presence of other resistance mechanisms. For instance, in *E. coli*, the 16S-RMTase RmtB was found to correlate the best with meropenem resistance as the two isolates that produced RmtB also produced NDM. Similarly, aminoglycoside resistant *E. coli* isolates often produced TEM and OXA-1, while ciprofloxacin resistant *K. pneumoniae* isolates often produced TEM as well.

**Table 5:** All protein groups which were more than four times as abundant in resistant *E. coli* isolates compared to susceptible *E. coli* isolates.

Protein	Most correlated to resistance to	P-value	Fold change	Curated in CARD
Class C extended-spectrum beta-lactamase CMY-42	3rd gen cephalosporins	< 0.001	6.13	Yes
Class A extended-spectrum beta-lactamase CTX-M-15	3rd gen cephalosporins	< 0.001	24.77	Yes
Carbapenem-hydrolyzing class D beta-lactamase OXA-48	Meropenem	< 0.001	8.48	Yes
16S rRNA (guanine(1405)-N(7))-methyltransferase RmtB1	Meropenem	< 0.001	5.47	Yes
Subclass B1 metallo-beta-lactamase NDM-5	Meropenem	< 0.001	61.22	Yes
YkgJ family cysteine cluster protein	Meropenem	< 0.001	4.71	No
Polysaccharide export protein	Meropenem	< 0.001	6.09	No
Pseudouridine-5′-phosphate glycosidase	Meropenem	< 0.001	5.20	No
H-NS histone family protein	Meropenem	< 0.001	14.60	Yes
Aminoglycoside N-acetyltransferase AAC(3)-IIa	Aminoglycosides	< 0.001	5.82	Yes
Class A beta-lactamase TEM-210	Aminoglycosides	< 0.001	7.62	Yes
Mph(A) family macrolide 2′-phosphotransferase	Aminoglycosides	< 0.001	13.38	Yes
OXA-1 family class D beta-lactamase	Aminoglycosides	< 0.001	13.52	Yes
sce7725 family protein	Aminoglycosides	< 0.001	4.14	No
Superoxide dismutase [Fe]	Aminoglycosides	< 0.001	7.01	No
Ketobutyrate formate-lyase/pyruvate formate-lyase	Aminoglycosides	< 0.001	5.67	No
Phosphoenolpyruvate-protein phosphotransferase PtsI	Aminoglycosides	< 0.001	4.89	Yes
50S ribosomal protein L1	Aminoglycosides	0.0019	4.51	No
Fluoroquinolone-acetylating aminoglycoside 6′-N-acetyltransferase AAC(6′)-Ib-cr5	Ciprofloxacin	< 0.001	12.10	Yes
NADP-dependent phosphogluconate dehydrogenase	Ciprofloxacin	< 0.001	6.89	No
NADP-dependent phosphogluconate dehydrogenase	Ciprofloxacin	< 0.001	5.12	No
UDP-glucose 6-dehydrogenase	Ciprofloxacin	< 0.001	4.60	No
Plasmid-partitioning protein SopA	Ciprofloxacin	< 0.001	5.11	No

**Table 6:** All protein groups which were more than 4 times as abundant in resistant *K. pneumoniae* isolates compared to susceptible *K. pneumoniae* isolates.

Protein	Most correlated to resistance to	P-value	Fold change	Curated in CARD
Aminoglycoside O-phosphotransferase APH(3″)-Ib	3rd gen cephalosporins	< 0.001	5.70	Yes
Class A extended-spectrum beta-lactamase CTX-M-15	3rd gen cephalosporins	< 0.001	41.51	Yes
Heat shock survival AAA family ATPase ClpK	3rd gen cephalosporins	0.002	4.00	No
Subclass B1 metallo-beta-lactamase NDM-1	Meropenem	< 0.001	7.29	Yes
Class A extended-spectrum beta-lactamase SHV-5	Meropenem	< 0.001	8.74	Yes
Type A-1 chloramphenicol O-acetyltransferase	Meropenem	< 0.001	9.69	Yes
Aminoglycoside O-phosphotransferase APH(3′)-Ia	Meropenem	< 0.001	10.97	Yes
Carbapenem-hydrolyzing class A beta-lactamase KPC-2	Meropenem	< 0.001	20.03	Yes
Ribonuclease E	Meropenem	< 0.001	4.75	No
Type I restriction endonuclease subunit R	Meropenem	< 0.001	4.70	No
Porin (not otherwise specified)	Meropenem	< 0.001	4.07	No
Trigger factor	Meropenem	< 0.001	6.31	No
Hypothetical protein	Meropenem	< 0.001	4.72	No
Outer membrane protein assembly factor BamE	Meropenem	< 0.001	4.19	No
C-Lysozyme inhibitor	Meropenem	< 0.001	4.86	No
Aldo/keto reductase family oxidoreductase	Meropenem	< 0.001	4.26	No
SAM-dependent DNA methyltransferase	Meropenem	< 0.001	4.73	No
DUF305 domain-containing protein	Meropenem	< 0.001	12.18	No
Aminoglycoside N-acetyltransferase AAC(3)-IIa	Aminoglycosides	< 0.001	6.05	Yes
Fluoroquinolone-acetylating aminoglycoside 6′-N-acetyltransferase AAC(6′)-Ib-cr5	Aminoglycosides	< 0.001	32.97	Yes
Multidrug efflux RND transporter periplasmic adaptor subunit OqxA	Ciprofloxacin	< 0.001	5.04	Yes
Class A broad-spectrum beta-lactamase TEM-1	Ciprofloxacin	< 0.001	9.10	Yes
LPS assembly lipoprotein LptE	Ciprofloxacin	< 0.001	4.12	No

In addition to the protein groups curated in CARD, 23 other protein groups were more than four times as abundant in isolates resistant to any of the antibiotic classes. The majority of these protein groups were variants of protein groups not correlated with resistance. Four protein groups were an exception and had little to no variant groups. These were the YkgJ family cysteine cluster protein, the sce7725 family protein and the plasmid-partitioning protein SopA in *E. coli*, and the outer membrane protein assembly factor BamE in *K. pneumoniae*.

## Discussion

A thorough understanding of antimicrobial resistance is key for both the development of antibiotics and diagnostic tools. Antimicrobial resistance is mostly studied using growth-inhibition methods and DNA detection and sequencing techniques. However, as new AMR protein detection methods are developed that are based on MS^9,15,16^, it is important to know which resistance mechanisms can be detected at the protein level and if they can predict phenotypic resistance as well. In the current study, we performed a systematic analysis of meropenem, third generation cephalosporin, aminoglycoside, and ciprofloxacin resistance in 187 selected *E. coli* and *K. pneumoniae* isolates harboring different antibiotic resistance mechanisms. The majority of the studied isolates was unique as was determined by means of cgMLST, which suggests that our findings are generalizable for both bacterial species. We demonstrated that the proteins of different antimicrobial resistance mechanisms can be detected using a proteogenomic approach with bottom-up LC–MS/MS. Especially beta-lactamases, 16S-RMTases, and AACs were detected in the proteome with high sensitivity. Remarkably, some other mechanisms were not detected at all with LC–MS/MS, or they were only detected in a minority of the isolates with an encoding gene. For some AMR mechanisms, such as OXA-9-like, CMY-132-like or DHA, this could be explained by the presence of partial genes which did not lead to functional proteins and did also not confer resistance. Such genes might not be transcribed, or the resulting proteins might be degraded at an early stage. Other proteins which were not detected or only in a few isolates were aadA5, APH(6)-Id-like and QnrS. The low detection rate of these mechanisms might imply that the proteins are present in quantities below the detection limit, or that they are altered by post translational modifications. Alternatively, the encoding genes might only be transcribed and translated under certain conditions.

Although not all AMR mechanisms were detected at the protein level, resistance could be explained by the detected proteins for most of the selected isolates. In all isolates resistant to meropenem, a carbapenemase was detected. Furthermore, in all isolates resistant to 3rd generation cephalosporins, ESBLs, AmpC enzymes and/or carbapenemases were detected. Gentamicin and tobramycin resistance could be explained by the proteogenomic approach in 100% and 97% of the *E. coli* isolates, respectively. These numbers were lower for *K. pneumoniae*, the resistant phenotype could be explained in 89% of the gentamicin resistant isolates and 76% of the tobramycin resistant isolates. The AMR gene that resulted in tobramycin resistance could not be identified in 12 K*. pneumoniae* isolates even though AAC(6′)-Ib(-like) proteins were detected in nine of these isolates after additional analysis. A possible explanation for this discrepancy could be the use of only short-read sequencing resulting in incomplete coverage of genomes. This could also explain why *bla*_AmpC_ and *bla*_SHV_ genes were not detected in all of the *E. coli* and *K. pneumoniae* isolates^20,25^. Still, more than 95% of the core genes were detected in each isolate. Ciprofloxacin resistance in the studied isolates could be explained by mutations in *gyrA* and *parC*, and by the presence of AAC(6′)-Ib-cr enzymes, OqxAB efflux pumps and *qnr* genes. In the current study, we did not correlate mutations at the DNA level to the detected peptides as a targeted MS approach is more suitable to detect these key amino acid substitutions as demonstrated by Hassing et al.^26^.

In addition to the analysis of resistance mechanisms curated in CARD, we analyzed porin abundance as a decrease in porins or complete loss of a porin contributes to resistance^27^. For instance, a total lack of OmpC is associated with resistance to quinolones^28^, and beta-lactams^27,29^. This was not demonstrated in the current study but instead a variant of OmpC was detected that was significantly less abundant in *E. coli* isolates resistant to 3rd generation cephalosporins, aminoglycosides or ciprofloxacin. This OmpC variant differed substantially compared to the “regular” OmpC and differences in multiple regions may affect membrane permeability. For instance, the insertion of Gln in loop L3 (position 142 of the alignment) could affect the pore diameter^27^. Additional experiments are required to assess which of the structural differences affect permeability to antibiotics the most. Furthermore, we demonstrated that both OmpF in *E. coli* and its orthologue OmpK35 in *K. pneumoniae* were less abundant in resistant isolates. This finding corresponds with previous literature in which the absence of OmpF, or expression of the less permeable OmpC instead of OmpF resulted in a reduced influx of beta-lactams and quinolones and a resulting increase in MICs^10,27,30,31^.

Furthermore, a discovery-based analysis was performed by which we identified several correlations of proteins to resistance. We found that the plasmid-partitioning protein SopA was significantly more abundant in ciprofloxacin resistant *E. coli* isolates. This protein plays a role in plasmid partitioning of F plasmids which are major carriers of acquired resistance genes in *E. coli*^32,33^. In *K. pneumoniae*, presence of the outer membrane protein assembly factor BamE was significantly correlated to resistance. Previously, Sikora et al. described BamE was not a vital protein for *N. gonorrhoeae*, but absence of BamE resulted in an altered cell envelope composition and an increase in antibiotic susceptibility^34^. We did not find links in the literature between antimicrobial resistance and the identified YkgJ family cysteine cluster protein or the sce7725 family protein. Nonetheless, none of the genes encoding these four proteins were located next to genes of curated AMR mechanisms indicating a (independent) correlation to resistance in the studied isolates. In addition to these protein groups, many other protein groups were identified that correlated to resistance (“Supplementary datasets”). Further experiments and analyses are required to assess if and how these protein groups are involved in antibiotic resistance.

Previous studies which applied discovery based-proteomics in *E. coli* and *K. pneumoniae* analyzed a single or a few isolates^35–38^, or focused on a specific mechanism^39^. In the current study, resistance against commonly used antibiotics within the beta-lactam, aminoglycoside, and fluoroquinolone groups was analyzed in a significant number of clinical *E. coli* and *K. pneumoniae* isolates by a combination of both LC–MS/MS and WGS. Altogether, our findings indicate that the majority of the known AMR proteins causing resistance to beta-lactams and aminoglycosides can be detected by bottom-up proteomics without prior exposure to antibiotics. The high detection rate of resistance mechanisms by MS was facilitated by the use of a protein database which was assembled using WGS data from all of the studied isolates. Unfortunately, publicly available protein databases such as UniProt are incomplete for resistance mechanisms and require the inclusion of proteins from many bacterial species which results in more false-positives and/or a decrease in sensitivity. The quality of the protein database affected the sensitivity and specificity of the current LC–MS/MS analyses and shows its evident potential provided that a concise and optimal database is used. The current findings supports research endeavors aiming to develop rapid protein detection methods for antimicrobial resistance testing, perhaps by using a shorter sample pre-treatment protocol and a more high-throughput LC–MS method. Furthermore, the extensive amount of multi-omics data generated in this study showed correlations between resistance and various proteins that were not yet described. Finally, this study shows LC–MS/MS is a different and complementary method which can be used to study antimicrobial resistance in detail.

## Materials and methods

### Bacterial isolates

Ethical approval was not required, as only stored bacterial isolates were used. A variety of different *E. coli* and *K. pneumoniae* isolates were obtained with most of the isolates being resistant to one or more of the antibiotics of interest. Altogether, 187 isolates were used of which 117 *E. coli* and *K. pneumoniae* isolates were obtained from the Erasmus MC collection. Of these, nine *E. coli* isolates were selected based on a phenotype which suggested hyperproduction of chromosomal AmpC (MICs for cefoxitin > 16 mg/L, MICs for ceftazidime of 4–8 mg/L and twofold higher than MICs for ceftriaxone)^40^. Furthermore, 10 *E. coli* isolates were selected that were known to carry a CMY gene and 23 *E. coli* and *K. pneumoniae* isolates were selected based on being resistant to gentamicin, ciprofloxacin or both. The remaining 75 isolates were either carbapenem resistant and/or 3rd generation cephalosporin resistant, or they were susceptible to both carbapenems and 3rd generation cephalosporins. In addition, 46 *E. coli* and *K. pneumoniae* isolates that were ESBL and/or carbapenemase-positive were obtained from the Dutch National Institute for Public Health and the Environment (RIVM), 13 predominantly VIM positive *K. pneumoniae* isolates were obtained from the National School of Public Health, Athens, Greece, and 11 predominantly OXA-48 positive *K. pneumoniae* isolates were obtained from the Regional Institute of Gastroenterology and Hepatology in Cluj Napoca, Romania. The MICs of all isolates are displayed in Supplementary Tables 1 and 2.

### Culture protocol

Sub-cultured isolates stored at − 80 °C were thawed, cultured on Trypticase™ Soy Agar II plates with 5% sheep blood (Becton Dickinson, New Jersey, USA), and incubated overnight at 37 °C. Subsequently, one inoculation loop of bacteria was inoculated in 30 mL MH II broth and incubated overnight at 37 °C at 150 rpm. Next, the broth culture was centrifuged for 30 min at 4500*g*, and the pellet was washed with 10 mL phosphate-buffered saline. Subsequently, 6 mL phosphate-buffered saline was added, the samples were vortexed and 1 mL was transferred in each of six aliquots which were centrifuged for 5 min at 21,000*g*. The resulting six identical pellets per isolate were stored at − 80 °C. These pellets were used for AST, WGS and LC–MS/MS.

### Identification and AST

All isolates were previously identified in our laboratory using the MALDI biotyper (Bruker, Billerica, USA). AST was performed using custom microdilution Sensititre^®^ MIC susceptibility plates in accordance with the manufacturer’s instructions (Thermo Fisher Scientific, Waltham, United States). Clinical breakpoints and ECOFFs of the EUCAST were applied for the general classification of the isolates. For the discovery-based analysis which included the porin analysis, different breakpoints were used which were closer to the ECOFFs to detect protein abundance differences resulting in modest MIC changes. Both EUCAST breakpoints and the selected breakpoints are shown in Supplementary Table 3.

### Whole genome sequencing

DNA isolation and whole genome sequencing of all isolates was performed by Microsynth AG (Balgach, Switzerland). Extraction, lysis and isolation was performed using the Fast DNA Stool Mini Kit (Qiagen, Hilden, Germany). Bead beating was done in 2 mL screwcap tubes containing 0.6 g 0.1 mm glass beads by a FastPrep-24™ instrument using four cycles of 45 s (MP Biomedicals, Santa Ana, United States). Subsequently, 200 µL raw extract was prepared for DNA-isolation. The isolated DNA concentration was assessed with PicoGreen measurement (Quant-iT™ PicoGreen™ dsDNA Assay Kit, Thermo Fisher Scientific, Waltham, United States). For whole genome sequencing Illumina Nextera XT libraries were prepared using the Nextera XT DNA Library preparation kit (Illumina, San Diego, United states) according to manufacturer’s instructions. Subsequently, the libraries were checked for quality and library size on a 2100 Bioanalyzer instrument using a High Sensitivity DNA Assay kit (Agilent Technologies, Santa Clara, United States). The final libraries were quantified using a Quant-iT™ PicoGreen™ ds DNA Assay Kit (Thermo Fisher Scientific, Waltham, United States) and equimolarly pooled prior to sequencing. Sequencing was performed on an Illumina NextSeq 500/550 sequencing system using a NextSeq 500/550 High Output Kit v2.5 for 300 cycles (Illumina, San Diego, United States). The produced 2 × 150 bp paired-end reads passing Illumina’s chastity filter were demultiplexed with the software bcl2fastq (version 2.19.1.403, Illumina, San Diego, United States) allowing a maximum of one mismatch per index. Quality of the sequencing reads in fastq format was checked with the software FastQC (version 0.11.7). Genomes were assembled using Unicycler v0.4 with default parameters^41^.

### Reagents for lysis and digestion prior to LC–MS/MS

All reagents were provided by Thermo Fisher Scientific (Waltham, United States) unless otherwise specified. Dithiothreitol (DTT) and iodoacetamide (IAA) were dissolved in 50 mM ammonium bicarbonate (Sigma-Aldrich, St. Louis, United States). The digest buffer contained 50 mM triethylammonium bicarbonate buffer, 5% acetonitrile (Lab-Scan, Gliwice, Poland) and 0.5% w/w sodium deoxycholate (Sigma-Aldrich, St. Louis, United States) in water. The lysis buffer was identical to the digest buffer except sodium deoxycholate ratio was 5% w/w and DTT was added to a final concentration of 7.5 mM in the lysis buffer. TPCK treated trypsin was provided by Worthington (New Jersey, USA) and was dissolved in 50 mM formic acid, final concentration 1 μg/μL.

### Sample preparation LC–MS/MS

To prepare samples for LC–MS/MS, bacterial pellets were lysed in 300 μL lysis buffer and incubated for 10 min at 80 °C and 450 rpm (Eppendorf ThermoMixer^®^ C, Eppendorf, Hamburg, Germany). Lysates were sonicated for 2 min, 70% amplitude on a Digital Sonifier^®^ (Branson, Danbury, USA). Subsequently, 50 μL was transferred to a new tube and 22.5 μL DTT (50 mM) was added followed by 20 min of incubation at 60 °C and 450 rpm. Samples were cooled to room temperature and alkylated with 25 μL IAA (100 mM) followed by 30 min incubation in the dark at room temperature. Subsequently, proteins were precipitated by washing twice with 900 μl acetone stored at − 20 °C (Sigma-Aldrich, St. Louis, United States). Next, 100 μL digest buffer was added and samples were sonicated for 90 s, 70% amplitude to dissolve the pellet. Ten μL trypsin solution was added and samples were incubated overnight. To stop the reaction 17 μL 5% TFA in water was added and samples were centrifuged for 10 min (21,000*g*, 4 °C). Supernatant was transferred to a new tube. A Pierce™ Quantitative Colorimetric Peptide Assay was used to analyze the protein concentration of all digests. All samples were vacuum dried (Savant™ SpeedVac™ Concentrator, Thermo Fisher Scientific, Waltham, United States) and stored at − 80 °C.

### LC–MS/MS

Digests were diluted with 0.1% trifluoracetic acid to a final peptide concentration of 200 ng/μL. Five μL of each injected sample was analyzed using a nano-LC (Ultimate 3000RS, Thermo Fisher Scientific, Germering, Germany). After preconcentration and washing the samples on a C18 trap column (1 mm × 300 μm internal diameter (ID), Thermo Fisher Scientific), samples were loaded onto a C18 column (PepMap C18, 75 µm ID × 250 mm, 2 μm particle and 100 Å pore size, Thermo Fisher Scientific) using a linear 90-min gradient (4–38% ACN/H20; 0.1% formic acid) at a flow rate of 300 nL/min. The separation of the peptides was monitored by a UV detector (absorption at 214 nm). The nano-LC was coupled to an Orbitrap Fusion Lumos (Thermo Fisher Scientific, San Jose, CA, USA). The Orbitrap Fusion Lumos was operated in data dependent acquisition (DDA) mode. Full scan MS spectra (m/z 375–1500) in profile mode were acquired in the Orbitrap with a resolution of 120,000 after accumulation of an AGC target of 400,000. A top speed method with a maximum duty cycle of 3 s was used. In these 3 s the most intense peptide ions from the full scan in the Orbitrap were fragmented by HCD (normalized collision energy 30%) and measured in the iontrap with a AGC target of 10,000. Maximum fill times were 50 ms for the full scans and 50 ms for the MS/MS scans. Precursor ion charge state screening was enabled and only charge states from 2 to 7 were selected for fragmentation. Dynamic exclusion was activated after the first time a precursor was selected for fragmentation and excluded for a period of 60 s using a relative mass window of 10 ppm. Lock mass correction was activated to improve mass accuracy of the survey scan. After each measurement the column was rinsed with a blank to minimize carry-over. After each ten samples a quality control was measured to assess shifting retention times and data quality.

### WGS data analysis

WGS data was first annotated with Prokka v1.13^42^. All genomes were analyzed to identify curated AMR genes using a stand-alone version of the Resistance Gene Identifier (RGI) v5.1.0 based on the Comprehensive Antibiotic Resistance Database (CARD) v3.0.5^43^. Only perfect and strict hits were allowed. COG analysis^18^ was performed with WebMGA^44^. Statistics and visualizations were performed in R^45^. For *E. coli* and *K. pneumoniae,* cgMLST was performed using SeqSphere (Münster, Germany) and available core gene sets of 2513 genes for *E. coli* and 2358 genes for *K. pneumonia*. BioNumerics (Applied Maths, Sint-Martens-Latem, Belgium) was used to depict clustering of isolates by minimum spanning trees.

### DDA data processing and analysis

MaxQuant 1.6.1.0 (Max Planck Institute for Chemistry, Mainz, Germany) was used to analyze the DDA data; default settings were used unless indicated otherwise. A maximum of two missed cleavages was allowed. Oxidation was set as a variable modification of methionine, carbamidomethylation as a fixed modification of cysteine, and trypsin was set as enzyme. The used protein database was assembled using the WGS data which was first annotated using Prokka v1.13^42^. However, as many proteins were annotated as hypothetical proteins, annotation was repeated using the protein sequences of all bacteria and plasmid entries of the NCBI RefSeq database (29th of April 2020, 138,661,652 entries). This was performed using the protein–protein basic local alignment search tool (blastp, version 2.6.0^46^). In MaxQuant, the label free quantitation option with matching between runs was used. The quantitative values for all identified protein groups were further analyzed in Perseus 1.6.1.2 (Max Planck Institute for Chemistry, Mainz, Germany). The data were annotated, log2 transformed, and imputation was applied for missing values (in which missing values were replaced by values from a down shifted normal distribution of the intensities). Subsequently, hierarchical clustering and statistical analyses were performed to generate significance tables and protein intensity tables. To identify proteins of which the abundance was significantly correlated to resistance, a volcano plot analysis was performed based on unpaired t-tests. The cut-off for significance was based on 250 randomizations of the data set and was set at a false discovery rate of 5%. Proteins of AMR mechanisms curated in CARD were analyzed separately. These mechanisms were considered present when the encoding gene was present and at least one specific peptide of the protein was detected. Porin intensity plots were made using GraphPad Prism (GraphPad Software, San Diego, USA). OmpC variant sequences were compared using Clustal W version 2.1^47^. Similar to the analysis of WGS data, WebMGA and BioNumerics were used for COG analysis and clustering, respectively.

### Transparency declarations

The ErasmusMC is patent holder of “mass spectrometric determination of drug resistance” (PCT/NL2013/050255) which is licensed to Da Vinci Laboratory Solutions (Rotterdam, the Netherlands).

## Supplementary Information

Supplementary Information 1.

Supplementary Information 2.

Supplementary Figure 1.

Supplementary Information 3.

Supplementary Information 4.

Supplementary Information 5.

## Data Availability

The genomic sequencing data of the 187 *E. coli* and *K. pneumoniae* isolates are available in the ENA repository under the primary accession number PRJEB41042 and secondary accession number ERP124768. The mass spectrometry proteomics data have been deposited to the ProteomeXchange Consortium via the PRIDE^48^ partner repository with the dataset identifier PXD023736 for the *E. coli* data and PXD023739 for the *K. pneumoniae* data. All other data is included in this article or as “Supplemental material”.

## References

[1] WHO. *Antimicrobial resistance: Global report on surveillance 2014*. https://www.who.int/drugresistance/documents/surveillancereport/en/ (2014).

[2] Ahmed M (2018). Acute cholangitis—An update. World J. Gastrointest. Pathophysiol..

[3] Laupland KB (2013). Incidence of bloodstream infection: A review of population-based studies. Clin. Microbiol. Infect..

[4] Cassini A (2019). Attributable deaths and disability-adjusted life-years caused by infections with antibiotic-resistant bacteria in the EU and the European Economic Area in 2015: A population-level modelling analysis. Lancet Infect. Dis..

[5] WHO. Global priority list of antibiotic-resistant bacteria to guide research, discovery, and development of new antibiotics. https://www.who.int/medicines/publications/global-priority-list-antibiotic-resistant-bacteria/en/ (2017).

[6] Stoesser N (2013). Predicting antimicrobial susceptibilities for *Escherichia coli* and *Klebsiella pneumoniae* isolates using whole genomic sequence data. J. Antimicrob. Chemother..

[7] Consortium, C.R. (2018). Prediction of Susceptibility to first-line tuberculosis drugs by DNA sequencing. N. Engl. J. Med..

[8] Gordon NC (2014). Prediction of *Staphylococcus aureus* antimicrobial resistance by whole-genome sequencing. J. Clin. Microbiol..

[9] Charretier Y, Schrenzel J (2016). Mass spectrometry methods for predicting antibiotic resistance. Proteom. Clin. Appl..

[10] Wan Nur Ismah WAK (2018). Prediction of fluoroquinolone susceptibility directly from whole-genome sequence data by using liquid chromatography-tandem mass spectrometry to identify mutant genotypes. Antimicrob. Agents Chemother..

[11] Martinez-Martinez L (2008). Extended-spectrum beta-lactamases and the permeability barrier. Clin. Microbiol. Infect..

[12] Hebert AS (2014). The one hour yeast proteome. Mol. Cell Proteom..

[13] Chang CJ (2013). Diagnosis of beta-lactam resistance in *Acinetobacter baumannii* using shotgun proteomics and LC-nano-electrospray ionization ion trap mass spectrometry. Anal. Chem..

[14] Trip H (2015). Simultaneous identification of multiple beta-lactamases in *Acinetobacter baumannii* in relation to carbapenem and ceftazidime resistance, using liquid chromatography-tandem mass spectrometry. J. Clin. Microbiol..

[15] Welker M, van Belkum A (2019). One system for all: Is mass spectrometry a future alternative for conventional antibiotic susceptibility testing?. Front. Microbiol..

[16] Foudraine DE (2019). Accurate detection of the four most prevalent carbapenemases in *E. coli* and *K. pneumoniae* by high-resolution mass spectrometry. Front. Microbiol..

[17] van Belkum A (2020). Innovative and rapid antimicrobial susceptibility testing systems. Nat. Rev. Microbiol..

[18] Tatusov RL, Galperin MY, Natale DA, Koonin EV (2000). The COG database: A tool for genome-scale analysis of protein functions and evolution. Nucleic Acids Res..

[19] Jacoby GA (2009). AmpC beta-lactamases. Clin. Microbiol. Rev..

[20] Livermore DM (1995). beta-Lactamases in laboratory and clinical resistance. Clin. Microbiol. Rev..

[21] Ramirez MS, Tolmasky ME (2010). Aminoglycoside modifying enzymes. Drug Resist. Updat..

[22] Jacoby GA (2006). Beta-lactamase nomenclature. Antimicrob. Agents Chemother..

[23] Doi Y, Wachino JI, Arakawa Y (2016). Aminoglycoside resistance: The emergence of acquired 16S ribosomal RNA methyltransferases. Infect. Dis. Clin. N. Am..

[24] van der Putten BCL (2019). Quantifying the contribution of four resistance mechanisms to ciprofloxacin MIC in *Escherichia coli*: A systematic review. J. Antimicrob. Chemother..

[25] Babini GS, Livermore DM (2000). Are SHV beta-lactamases universal in *Klebsiella pneumoniae*?. Antimicrob. Agents Chemother..

[26] Hassing RJ (2016). Detection of amino acid substitutions in the GyrA protein of fluoroquinolone-resistant typhoidal Salmonella isolates using high-resolution mass spectrometry. Int. J. Antimicrob. Agents.

[27] Vergalli J (2020). Porins and small-molecule translocation across the outer membrane of Gram-negative bacteria. Nat. Rev. Microbiol..

[28] Chenia HY, Pillay B, Pillay D (2006). Analysis of the mechanisms of fluoroquinolone resistance in urinary tract pathogens. J. Antimicrob. Chemother..

[29] Choi U, Lee CR (2019). Distinct roles of outer membrane porins in antibiotic resistance and membrane integrity in *Escherichia coli*. Front. Microbiol..

[30] Masi M, Refregiers M, Pos KM, Pages JM (2017). Mechanisms of envelope permeability and antibiotic influx and efflux in Gram-negative bacteria. Nat. Microbiol..

[31] Correia S, Poeta P, Hebraud M, Capelo JL, Igrejas G (2017). Mechanisms of quinolone action and resistance: Where do we stand?. J. Med. Microbiol..

[32] Mori H, Kondo A, Ohshima A, Ogura T, Hiraga S (1986). Structure and function of the F plasmid genes essential for partitioning. J. Mol. Biol..

[33] Stephens C (2020). F plasmids are the major carriers of antibiotic resistance genes in human-associated commensal *Escherichia coli*. mSphere.

[34] Sikora AE (2018). Structural and functional insights into the role of BamD and BamE within the beta-barrel assembly machinery in *Neisseria gonorrhoeae*. J. Biol. Chem..

[35] Soufi B, Krug K, Harst A, Macek B (2015). Characterization of the *E. coli* proteome and its modifications during growth and ethanol stress. Front. Microbiol..

[36] Sharma D, Garg A, Kumar M, Rashid F, Khan AU (2019). Down-regulation of flagellar, fimbriae, and pili proteins in carbapenem-resistant *Klebsiella pneumoniae* (NDM-4) clinical isolates: A novel linkage to drug resistance. Front. Microbiol..

[37] Keasey SL (2019). Decreased antibiotic susceptibility driven by global remodeling of the *Klebsiella pneumoniae* proteome. Mol. Cell Proteom..

[38] Uddin MJ, Ma CJ, Kim JC, Ahn J (2019). Proteomics-based discrimination of differentially expressed proteins in antibiotic-sensitive and antibiotic-resistant *Salmonella typhimurium*, *Klebsiella pneumoniae*, and *Staphylococcus aureus*. Arch. Microbiol..

[39] Cudic E, Surmann K, Panasia G, Hammer E, Hunke S (2017). The role of the two-component systems Cpx and Arc in protein alterations upon gentamicin treatment in *Escherichia coli*. BMC Microbiol..

[40] Paltansing S (2015). Increased expression levels of chromosomal AmpC beta-lactamase in clinical *Escherichia coli* isolates and their effect on susceptibility to extended-spectrum cephalosporins. Microb. Drug Resist..

[41] Wick RR, Judd LM, Gorrie CL, Holt KE (2017). Unicycler: Resolving bacterial genome assemblies from short and long sequencing reads. PLoS Comput. Biol..

[42] Seemann T (2014). Prokka: Rapid prokaryotic genome annotation. Bioinformatics.

[43] Alcock BP (2020). CARD 2020: Antibiotic resistome surveillance with the comprehensive antibiotic resistance database. Nucleic Acids Res..

[44] Wu S, Zhu Z, Fu L, Niu B, Li W (2011). WebMGA: A customizable web server for fast metagenomic sequence analysis. BMC Genom..

[45] R Core Team (2020). A Language and Environment for Statistical Computing.

[46] National Center for Biotechnology Information (US). BLAST® Command Line Applications User Manual. https://www.ncbi.nlm.nih.gov/books/NBK279684/ (2018).

[47] Larkin MA (2007). Clustal W and Clustal X version 2.0. Bioinformatics.

[48] Perez-Riverol Y (2019). The PRIDE database and related tools and resources in 2019: Improving support for quantification data. Nucleic Acids Res..

