# Supplementary figures and images for: Exploring antimicrobial resistance to beta-lactams, aminoglycosides and fluoroquinolones in E. coli and K. pneumoniae using proteogenomics
Source: Sci Rep. 2021 Jun 14;11:12472. doi: 10.1038/s41598-021-91905-w (PMC8203672; doi:10.1038/s41598-021-91905-w)

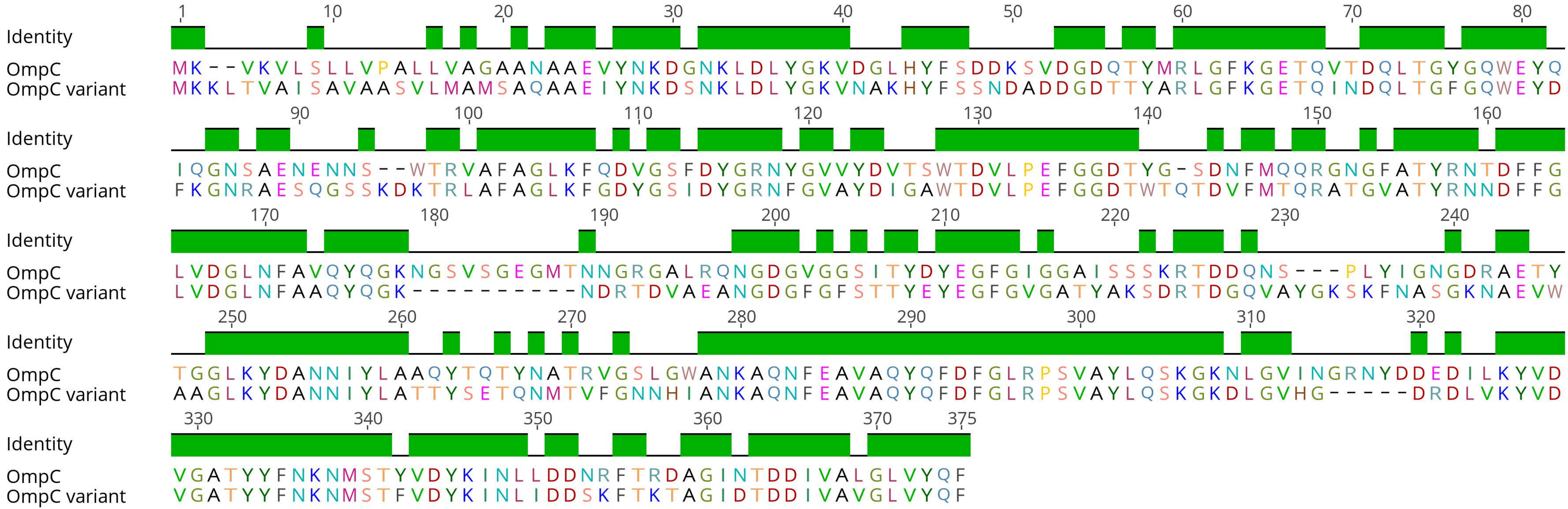

Supplement: Supplementary file 3 — Supplementary Figure 1. [file 41598_2021_91905_MOESM3_ESM.pdf]
