# Supplementary material for: Exploring antimicrobial resistance to beta-lactams, aminoglycosides and fluoroquinolones in E. coli and K. pneumoniae using proteogenomics
Source: Sci Rep. 2021 Jun 14;11:12472. doi: 10.1038/s41598-021-91905-w (PMC8203672; doi:10.1038/s41598-021-91905-w)
Supplement: Supplementary file 4 — Supplementary Information 3. [file 41598_2021_91905_MOESM4_ESM.docx]

**Supplementary table 1.** Minimum inhibitory concentrations of the 78 *E. coli* used in this study.

| Isolate number | Minimum inhibitory concentrations (mg/L) | | | | | |
| --- | --- | --- | --- | --- | --- | --- |
|  | Ceftriaxone | Ceftazidime | Meropenem | Gentamicin | Tobramycin | Ciprofloxacin |
| 1 | >8 | 8 | ≤0.06 | 1 | 1 | >4 |
| 2 | >8 | >8 | ≤0.06 | >8 | >8 | >4 |
| 3 | >8 | 4 | ≤0.06 | >8 | >8 | >4 |
| 4 | >8 | 8 | ≤0.06 | 2 | >8 | >4 |
| 5 | >8 | >8 | ≤0.06 | >8 | >8 | >4 |
| 6 | >8 | 2 | ≤0.06 | 1 | 1 | >4 |
| 7 | >8 | 4 | ≤0.06 | 1 | 1 | ≤0.06 |
| 8 | >8 | 4 | ≤0.06 | 1 | >8 | >4 |
| 9 | >8 | >8 | ≤0.06 | >8 | >8 | >4 |
| 10 | >8 | 4 | ≤0.06 | ≤0.5 | ≤0,5 | ≤0.06 |
| 11 | ≤0.25 | ≤0.25 | ≤0.06 | 2 | 1 | >4 |
| 12 | ≤0.25 | ≤0.25 | ≤0.06 | 1 | 1 | 0.25 |
| 13 | ≤0.25 | ≤0.25 | ≤0.06 | ≤0.5 | 1 | ≤0.06 |
| 14 | ≤0.25 | 0.5 | ≤0.06 | 1 | ≤0,5 | ≤0.06 |
| 15 | ≤0.25 | ≤0.25 | ≤0.06 | ≤0.5 | ≤0,5 | ≤0.06 |
| 16 | ≤0.25 | ≤0.25 | ≤0.06 | 1 | 1 | ≤0.06 |
| 17 | ≤0.25 | ≤0.25 | ≤0.06 | 1 | 1 | ≤0.06 |
| 18 | ≤0.25 | ≤0.25 | ≤0.06 | 1 | 1 | ≤0.06 |
| 19 | ≤0.25 | ≤0.25 | ≤0.06 | 1 | 2 | ≤0.06 |
| 20 | ≤0.25 | ≤0.25 | ≤0.06 | 1 | 1 | 0.25 |
| 48 | 0.5 | ≤0.25 | 1 | 1 | >8 | >4 |
| 60 | >8 | 4 | 8 | 1 | 1 | ≤0.06 |
| 63 | >8 | >8 | >32 | ≤0.5 | 1 | >4 |
| 64 | >8 | >8 | ≤0.06 | >8 | >8 | >4 |
| 67 | >8 | >8 | 16 | >8 | >8 | >4 |
| 70 | >8 | >8 | 32 | 1 | >8 | >4 |
| 71 | >8 | >8 | 16 | >8 | >8 | >4 |
| 73 | >8 | 4 | 2 | 1 | >8 | >4 |
| 78 | >8 | >8 | 4 | ≤0.5 | ≤0.5 | 0.25 |
| 80 | 0.5 | 0.5 | 0.5 | ≤0.5 | ≤0.5 | ≤0.06 |
| 86 | >8 | >8 | 8 | >8 | >8 | >4 |
| 106 | ≤0.25 | ≤0.25 | ≤0.06 | ≤0.5 | ≤0.5 | ≤0.06 |
| 118 | 2 | 2 | ≤0.06 | 1 | 1 | >4 |
| 119 | >8 | >8 | ≤0.06 | ≤0.5 | ≤0.5 | ≤0.06 |
| 120 | >8 | >8 | ≤0.06 | 1 | 1 | 0.12 |
| 121 | 8 | 8 | ≤0.06 | 1 | 2 | 0.25 |
| 122 | 8 | >8 | ≤0.06 | 1 | 1 | 0.25 |
| 123 | >8 | >8 | ≤0.06 | >8 | >8 | ≤0.06 |
| 124 | >8 | >8 | ≤0.06 | 1 | >8 | >4 |
| 125 | 8 | 8 | ≤0.06 | 1 | 1 | ≤0.06 |
| 126 | 8 | 8 | ≤0.06 | >8 | 4 | ≤0.06 |
| 127 | 4 | 8 | ≤0.06 | ≤0.5 | ≤0.5 | >4 |
| 128 | >8 | >8 | ≤0.06 | ≤0.5 | 1 | 0.12 |
| 140 | >8 | >8 | 0.12 | >8 | >8 | >4 |
| 141 | ≤0.25 | ≤0.25 | ≤0.06 | ≤0.5 | ≤0.5 | >4 |
| 142 | ≤0.25 | ≤0.25 | ≤0.06 | ≤0.5 | ≤0.5 | >4 |
| 143 | ≤0.25 | ≤0.25 | ≤0.06 | 1 | 1 | >4 |
| 144 | >8 | >8 | ≤0.06 | >8 | >8 | >4 |
| 145 | ≤0.25 | 0.5 | ≤0.06 | >8 | 8 | >4 |
| 146 | >8 | >8 | ≤0.06 | >8 | 8 | 1 |
| 147 | ≤0.25 | 0.5 | ≤0.06 | >8 | 4 | ≤0.06 |
| 148 | >8 | >8 | ≤0.06 | >8 | >8 | >4 |
| 149 | ≤0.25 | 0.5 | ≤0.06 | >8 | >8 | 2 |
| 150 | ≤0.25 | 0.5 | ≤0.06 | >8 | >8 | >4 |
| 151 | ≤0.25 | ≤0.25 | ≤0.06 | >8 | 8 | ≤0.06 |
| 152 | ≤0.25 | ≤0.25 | ≤0.06 | >8 | >8 | 0.25 |
| 153 | ≤0.25 | ≤0.25 | ≤0.06 | >8 | 8 | 0.12 |
| 154 | ≤0.25 | ≤0.25 | ≤0.06 | >8 | 4 | ≤0.06 |
| 171 | ≤0.25 | ≤0.25 | ≤0.06 | 1 | ≤0.5 | ≤0.06 |
| 172 | ≤0.25 | ≤0.25 | ≤0.06 | 1 | 1 | ≤0.06 |
| 173 | ≤0.25 | ≤0.25 | ≤0.06 | 1 | 1 | ≤0.06 |
| 174 | ≤0.25 | ≤0.25 | ≤0.06 | 1 | 1 | ≤0.06 |
| 175 | ≤0.25 | ≤0.25 | ≤0.06 | 1 | 1 | ≤0.06 |
| 176 | ≤0.25 | ≤0.25 | ≤0.06 | ≤0.5 | ≤0.5 | ≤0.06 |
| 177 | ≤0.25 | ≤0.25 | ≤0.06 | ≤0.5 | ≤0.5 | ≤0.06 |
| 178 | ≤0.25 | ≤0.25 | ≤0.06 | 1 | 1 | ≤0.06 |
| 179 | ≤0.25 | ≤0.25 | ≤0.06 | 1 | 1 | ≤0.06 |
| 180 | ≤0.25 | ≤0.25 | ≤0.06 | ≤0.5 | 1 | ≤0.06 |
| 181 | ≤0.25 | ≤0.25 | ≤0.06 | 1 | 1 | ≤0.06 |
| 182 | 2 | 8 | ≤0.06 | ≤0.5 | ≤0.5 | ≤0.06 |
| 183 | 0.5 | 4 | ≤0.06 | 1 | 1 | ≤0.06 |
| 184 | 2 | 8 | ≤0.06 | ≤0.5 | 1 | ≤0.06 |
| 185 | 0.5 | 4 | ≤0.06 | 2 | 8 | >4 |
| 186 | 1 | 4 | ≤0.06 | 2 | 2 | >4 |
| 187 | 4 | 8 | ≤0.06 | 1 | 1 | ≤0.06 |
| 188 | 2 | 8 | ≤0.06 | ≤0.5 | ≤0.5 | ≤0.06 |
| 189 | 1 | 4 | ≤0.06 | 1 | 1 | >4 |
| 190 | 1 | 4 | ≤0.06 | ≤0.5 | ≤0.5 | ≤0.06 |

**Supplementary table 2.** Minimum inhibitory concentrations of the 109 *K. pneumoniae* used in this study.

| Isolate number | Minimum inhibitory concentrations (mg/L) | | | | | |
| --- | --- | --- | --- | --- | --- | --- |
|  | Ceftriaxone | Ceftazidime | Meropenem | Gentamicin | Tobramycin | Ciprofloxacin |
| 21 | >8 | >8 | ≤0.06 | ≤0.5 | ≤0.5 | ≤0.06 |
| 22 | >8 | >8 | ≤0.06 | ≤0.5 | ≤0.5 | 0.5 |
| 23 | >8 | >8 | ≤0.06 | >8 | >8 | >4 |
| 24 | >8 | 1 | ≤0.06 | >8 | 4 | 1 |
| 25 | >8 | 4 | 0.12 | >8 | 2 | ≤0.06 |
| 26 | ≤0.25 | ≤0.25 | ≤0.06 | ≤0.5 | ≤0.5 | ≤0.06 |
| 27 | ≤0.25 | 0.5 | ≤0.06 | ≤0.5 | ≤0.5 | 4 |
| 28 | ≤0.25 | ≤0.25 | ≤0.06 | ≤0.5 | ≤0.5 | ≤0.06 |
| 29 | ≤0.25 | 1 | ≤0.06 | ≤0.5 | ≤0.5 | ≤0.06 |
| 30 | ≤0.25 | 0.5 | ≤0.06 | ≤0.5 | ≤0.5 | 1 |
| 32 | >8 | >8 | 16 | >8 | >8 | >4 |
| 33 | >8 | >8 | 2 | >8 | >8 | >4 |
| 34 | 2 | 1 | 4 | ≤0.5 | >8 | >4 |
| 35 | >8 | >8 | 32 | >8 | >8 | >4 |
| 36 | >8 | >8 | 4 | >8 | >8 | >4 |
| 37 | >8 | >8 | >32 | >8 | >8 | >4 |
| 38 | >8 | >8 | 1 | >8 | >8 | >4 |
| 39 | >8 | >8 | >32 | >8 | >8 | >4 |
| 40 | >8 | >8 | 1 | ≤0.5 | ≤0.5 | >4 |
| 41 | >8 | >8 | 0.5 | >8 | >8 | >4 |
| 42 | >8 | >8 | >32 | >8 | >8 | >4 |
| 43 | ≤0.25 | ≤0.25 | ≤0.06 | ≤0.5 | ≤0.5 | ≤0.06 |
| 44 | >8 | >8 | 4 | >8 | >8 | >4 |
| 45 | 0.5 | ≤0.25 | 0.5 | ≤0.5 | ≤0.5 | ≤0.06 |
| 46 | >8 | >8 | 4 | 2 | >8 | >4 |
| 47 | >8 | >8 | 4 | 2 | >8 | >4 |
| 49 | >8 | >8 | 4 | 2 | >8 | >4 |
| 50 | >8 | >8 | 4 | 2 | >8 | >4 |
| 51 | >8 | >8 | 1 | ≤0.5 | ≤0.5 | 0.5 |
| 52 | >8 | >8 | 32 | 1 | >8 | >4 |
| 53 | >8 | >8 | 4 | >8 | >8 | >4 |
| 54 | >8 | >8 | 1 | >8 | >8 | >4 |
| 55 | 0.5 | 0.5 | 0.25 | ≤0.5 | ≤0.5 | ≤0.06 |
| 56 | >8 | >8 | 8 | 4 | >8 | >4 |
| 57 | >8 | >8 | 4 | ≤0.5 | ≤0.5 | >4 |
| 58 | >8 | >8 | 16 | >8 | >8 | >4 |
| 59 | >8 | >8 | 4 | ≤0.5 | ≤0.5 | >4 |
| 61 | >8 | >8 | 0.5 | >8 | >8 | >4 |
| 62 | >8 | 8 | >32 | >8 | >8 | >4 |
| 65 | >8 | >8 | 4 | 2 | >8 | >4 |
| 66 | >8 | >8 | 8 | >8 | >8 | >4 |
| 68 | >8 | >8 | 0.5 | 1 | >8 | 1 |
| 69 | >8 | >8 | 1 | >8 | >8 | >4 |
| 72 | >8 | >8 | 8 | 2 | >8 | >4 |
| 74 | >8 | >8 | >32 | 4 | >8 | >4 |
| 75 | >8 | >8 | 4 | 2 | >8 | >4 |
| 76 | >8 | >8 | 4 | 2 | >8 | >4 |
| 77 | >8 | >8 | >32 | 4 | >8 | >4 |
| 79 | >8 | >8 | 8 | 2 | >8 | >4 |
| 81 | >8 | >8 | 16 | >8 | >8 | >4 |
| 82 | >8 | >8 | 2 | >8 | >8 | >4 |
| 83 | >8 | >8 | 1 | >8 | >8 | >4 |
| 84 | >8 | >8 | 16 | >8 | >8 | >4 |
| 85 | 1 | 1 | 1 | ≤0.5 | ≤0.5 | ≤0.06 |
| 87 | 0.5 | ≤0.25 | 1 | ≤0.5 | ≤0.5 | ≤0.06 |
| 88 | >8 | >8 | 8 | >8 | >8 | >4 |
| 89 | >8 | >8 | >32 | ≤0.5 | ≤0.5 | >4 |
| 90 | >8 | >8 | 4 | 1 | >8 | >4 |
| 91 | >8 | >8 | 4 | >8 | >8 | >4 |
| 92 | >8 | >8 | 32 | >8 | >8 | >4 |
| 93 | >8 | >8 | >32 | >8 | >8 | >4 |
| 94 | >8 | >8 | 16 | >8 | >8 | >4 |
| 95 | >8 | >8 | >32 | >8 | >8 | >4 |
| 96 | >8 | >8 | 8 | >8 | >8 | >4 |
| 97 | >8 | >8 | 32 | >8 | >8 | >4 |
| 98 | >8 | >8 | 16 | >8 | >8 | >4 |
| 99 | >8 | >8 | >32 | >8 | >8 | >4 |
| 100 | >8 | >8 | >32 | >8 | >8 | >4 |
| 101 | >8 | >8 | 32 | ≤0.5 | >8 | >4 |
| 102 | >8 | >8 | >32 | >8 | >8 | >4 |
| 103 | >8 | >8 | 0.5 | 1 | >8 | >4 |
| 104 | >8 | >8 | 4 | >8 | >8 | >4 |
| 105 | >8 | >8 | >32 | 2 | >8 | >4 |
| 107 | >8 | >8 | 16 | 1 | 8 | >4 |
| 108 | >8 | >8 | 16 | >8 | >8 | >4 |
| 109 | >8 | >8 | >32 | >8 | >8 | >4 |
| 110 | >8 | >8 | 1 | 1 | >8 | 0.12 |
| 111 | >8 | >8 | 8 | >8 | >8 | >4 |
| 112 | >8 | >8 | >32 | >8 | >8 | >4 |
| 113 | >8 | >8 | 1 | 8 | >8 | >4 |
| 114 | >8 | >8 | 2 | 1 | >8 | 0.12 |
| 115 | >8 | >8 | >16 | 8 | >8 | 1 |
| 116 | >8 | >8 | 4 | 1 | >8 | 0.25 |
| 117 | >8 | >8 | 4 | 4 | >8 | 2 |
| 129 | ≤0.25 | ≤0.25 | ≤0.06 | ≤0.5 | ≤0.5 | ≤0.06 |
| 130 | ≤0.25 | ≤0.25 | ≤0.06 | ≤0.5 | ≤0.5 | ≤0.06 |
| 131 | 0.5 | 0.5 | 1 | ≤0.5 | ≤0.5 | ≤0.06 |
| 132 | ≤0.25 | ≤0.25 | 0.12 | >8 | 1 | ≤0.06 |
| 133 | ≤0.25 | ≤0.25 | ≤0.06 | >8 | 1 | ≤0.06 |
| 134 | ≤0.25 | ≤0.25 | ≤0.06 | ≤0.5 | ≤0.5 | ≤0.06 |
| 135 | ≤0.25 | ≤0.25 | ≤0.06 | ≤0.5 | ≤0.5 | ≤0.06 |
| 136 | ≤0.25 | ≤0.25 | ≤0.06 | ≤0.5 | ≤0.5 | ≤0.06 |
| 137 | ≤0.25 | ≤0.25 | ≤0.06 | ≤0.5 | ≤0.5 | ≤0.06 |
| 155 | ≤0.25 | 1 | ≤0.06 | ≤0.5 | ≤0.5 | 0.25 |
| 156 | >8 | >8 | ≤0.06 | >8 | >8 | >4 |
| 157 | >8 | >8 | ≤0.06 | >8 | >8 | >4 |
| 158 | ≤0.25 | ≤0.25 | ≤0.06 | ≤0.5 | ≤0.5 | 0.5 |
| 159 | >8 | >8 | ≤0.06 | >8 | >8 | 2 |
| 160 | >8 | 8 | ≤0.06 | >8 | >8 | 2 |
| 161 | >8 | >8 | ≤0.06 | >8 | 8 | 0.5 |
| 162 | >8 | >8 | 0.25 | >8 | >8 | >4 |
| 163 | ≤0.25 | ≤0.25 | ≤0.06 | ≤0.5 | ≤0.5 | ≤0.06 |
| 164 | ≤0.25 | ≤0.25 | ≤0.06 | ≤0.5 | ≤0.5 | ≤0.06 |
| 165 | ≤0.25 | ≤0.25 | ≤0.06 | ≤0.5 | ≤0.5 | ≤0.06 |
| 166 | ≤0.25 | ≤0.25 | ≤0.06 | ≤0.5 | ≤0.5 | ≤0.06 |
| 167 | ≤0.25 | ≤0.25 | ≤0.06 | ≤0.5 | ≤0.5 | ≤0.06 |
| 168 | ≤0.25 | ≤0.25 | ≤0.06 | ≤0.5 | ≤0.5 | ≤0.06 |
| 169 | ≤0.25 | ≤0.25 | ≤0.06 | ≤0.5 | ≤0.5 | ≤0.06 |
| 170 | ≤0.25 | ≤0.25 | ≤0.06 | ≤0.5 | ≤0.5 | ≤0.06 |

**Supplementary table 3A and 3B.** Breakpoints for the classification of susceptible, intermediately susceptible and resistant isolates in mg/L. Table 3A shows the ECOFFs and clinical breakpoints from the EUCAST which were applied in this study. However, for the discovery-based analysis (including the porin analysis), different breakpoints were used which were closer to the ECOFFs to detect protein differences resulting in modest MIC changes (Table 3B). MIC’s in mg/L.

|  | ECOFF* | S** | R** |
| --- | --- | --- | --- |
| Ceftriaxone | 0.125 | ≤ 1 | ≥ 4 |
| Ceftazidime | 0.5 | ≤ 1 | ≥ 8 |
| Meropenem – *E. coli* | 0.064 | ≤ 2 | ≥ 16 |
| Meropenem – *K. pneumoniae* | 0.125 | ≤ 2 | ≥ 16 |
| Gentamicin | 2 | ≤ 2 | ≥ 4 |
| Tobramycin – *E. coli* | 4 | ≤ 2 | ≥ 4 |
| Tobramycin – *K. pneumoniae* | 2 | ≤ 2 | ≥ 4 |
| Ciprofloxacin – *E. coli* | 0.064 | ≤ 0.25 | ≥ 1 |
| Ciprofloxacin – *K. pneumoniae* | 0.125 | ≤ 0.25 | ≥ 1 |

**A.**

* ECOFF’s from the EUCAST (https://mic.eucast.org/Eucast2/ consulted on 09-10-2020)

** Clinical breakpoints from the EUCAST (https://www.eucast.org/clinical_breakpoints/ consulted on 09-10-2020)

|  | S | R |
| --- | --- | --- |
| Highest MIC of ceftazidime or ceftriaxone | ≤ 0.5 | ≥ 4 |
| Meropenem | ≤ 0.25 | ≥ 4 |
| Highest MIC of gentamicin or tobramycin | ≤ 1 | ≥ 4 |
| Ciprofloxacin | ≤ 0.12 | ≥ 1 |

**B.**

**Supplementary figure 1.** Amino acid sequences of the “regular” OmpC which was not significantly correlated to resistance and the OmpC variant which was.
